# Anodized Aluminum Oxide Membrane Ionic Memristors

**DOI:** 10.1021/jacs.4c16835

**Published:** 2025-03-20

**Authors:** Dipak Baram, Maksim Kvetny, Sarah Ake, Ruoyu Yang, Gangli Wang

**Affiliations:** Department of Chemistry, Georgia State University, Atlanta, Georgia 30302, United States

## Abstract

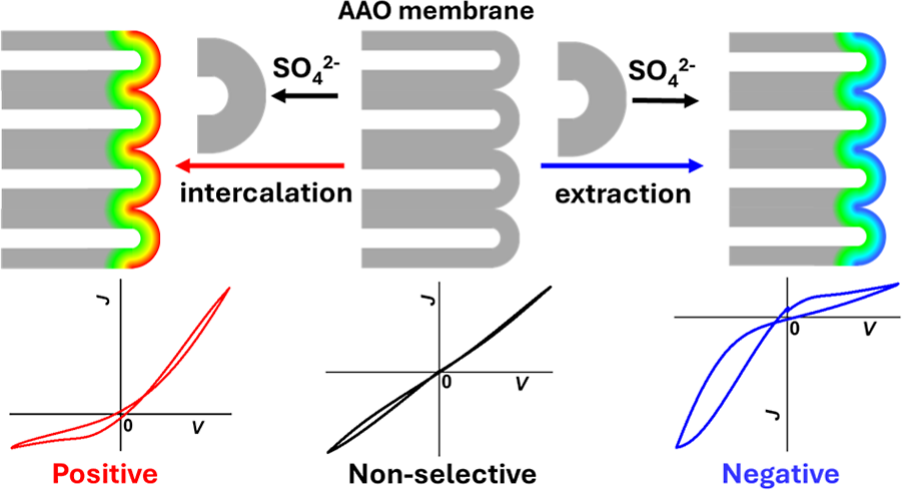

Memory effect in ion transport (IT) at the solid–solution interface is uniquely attractive in that the conductance depends on or “memorizes” the previous states. Hysteretic and rectified transport properties offer exciting potential to developing advanced iontronics and neuromorphic functions, improving the efficiency of energy conversion and electrochemical processes, and overcoming the selectivity-throughput bottleneck in the enrichment of low abundant species for environment- and energy-friendly separations, among others. Herein, memory effects are discovered in the rectified electrokinetic IT through anodized aluminum oxide (AAO) membranes containing densely packed highly ordered nanochannels (10^10^ per cm^2^). Characteristic memristor responses of pinched current–potential loops are resolved in voltammetric experiments and successfully reproduced through finite element simulation. Excitatory and inhibitory conductance states are shown to arise from the enrichment and depletion of mobile charge carriers. Structurewise, the transport symmetry is broken by the barrier oxide layer (BOL) on the one end of the cylindrical nanochannels across the AAO membranes. Charge selectivity is attributed to the gradient(s) of the space charge density across the BOL characterized by depth profiling via X-ray photoelectron spectroscopy analysis. The space charge gradient(s) overcomes the fundamental limitation of widely exploited surface charge effects to enable intense rectification and hysteresis prevailing at very high ionic concentrations up to 1–2 M. A new strategy is developed for controlling the preferential IT direction and selectivity via counterion intercalation and extraction/exchange. Mechanistic understanding is further confirmed through parameter variations such as potential scan rate and ionic strength, which also demonstrates convenient controls of the related functions.

## Introduction

Anodized aluminum oxide (AAO) membranes have attracted sustained research interest for decades with their densely packed well-ordered nanochannel array structures and rich physicochemical properties.^1−3^ Ion current rectification (ICR), a steady-state electrokinetic transport feature originally discovered in single conical nanopipettes, indicating preferential transport direction and selectivity,^4−8^ is recently observed in AAO membranes.^9,10^ Dynamic transport properties such as the intriguing memristor behaviors, hysteresis in the rectified ion transport (IT), and nonlinear dynamic current fluctuations^11−14^ remain to be demonstrated, much less on the mechanistic understanding or controls of the transport dynamics. Discovery of ICR in the easily accessible and structure-tunable macroscopic membranes, however, is already a significant step forward as those emerging nanostructure-confined transport properties offer new pathways for controlling the transport and storage of charges inaccessible in the bulk amorphous materials.^15−18^ Noteworthily, charge transport confined within nanoscale interface is often the exact physicochemical origin or a prominent factor in energy harvesting from salinity;^19,20^ in various electrochemistry energy conversion;^21,22^ in membrane and other separation systems especially for the challenging rare earth elements or other low abundant species that require pre-enrichment and high efficiency;^23−25^ in stochastic single entity sensing;^26,27^ and in the design and development of advance functions in iontronics and neuromorphic computation.^28−34^ In consideration that many applications are under nonequilibrium and nonsteady-state conditions and require more than a single nanopore,^35−37^ fundamental transport dynamics studies of IT through ensemble systems such as AAO membranes with broken transport symmetry are required to enable essential capabilities for advancing related applications.

**Scheme 1:**
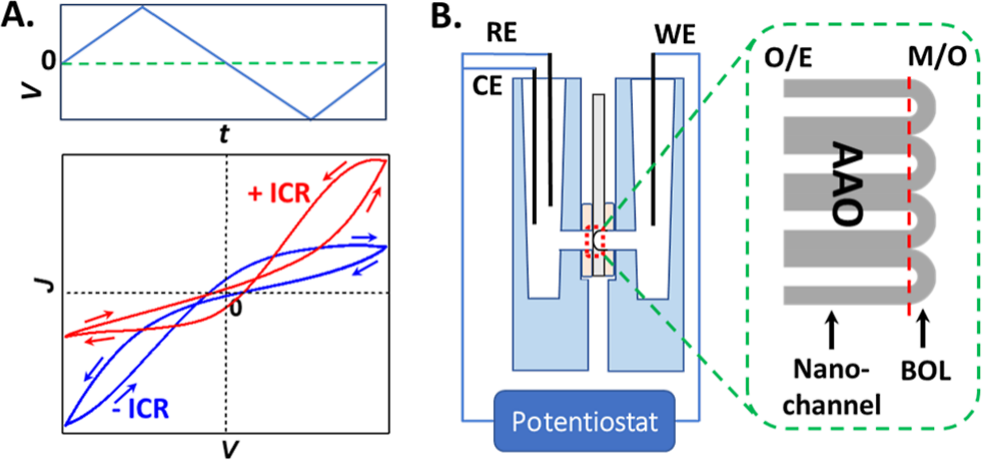
Characteristic Current–Potential (*I–V*) Responses under Triangular Potential-Time Wave (A) and Experimental Setup (B); Positive (Red) and Negative (Blue) ICR and Hysteresis Are Defined with Respect to High Conductivity State (HC) in the Positive Potential Range When the Working Electrode Is on the Barrier Oxide Layer (BOL) Side of the AAO, the Ground/Reference Electrode in the O/E Chamber; Arrows in the Current Density Plot Indicate the Directions of the Cyclic Potential Scans

Characteristic memristor features in pinched *I–V* loops are sketched in Scheme 1A. The loops can be perceived as two current branches with different ICR, which is the deviation from linear ohmic ionic conductance: current is higher at one potential polarity over the opposite at the same applied potential magnitude, referred to as high and low conductivity (HC/LC) states, respectively. ICR reflects the concentration polarization and inhomogeneous flux distribution, in other words more enriched and depleted mobile ion charge carriers. The localized surface electrical field facilitates one transport direction or type of ions, i.e., transport selectivity, when in alignment with the applied electrical field. Hysteretic loops emerge under varying potential stimulus, a time-dependent second-order derivative problem compared to ICR at the first order, displaying a characteristic cross-point away from origin (0,0) which separates at least two pinched current–potential loops.^38,39^ Those phenomena result from the history-dependent enrichment of mobile charge carriers at HC and depletion at LC, under the combined effects of the operational and intrinsic surface electrical fields.^10,11,40^ Similar to the ICR ratio, i.e., the current ratio at the same potential magnitude but opposite polarity, the ratio of the hysteresis charges within the HC and LC loops can serve as a convenient parameter to benchmark material-device properties. The governing factors from IT under nanoconfinements are an asymmetric structural confinement limiting the IT such as the conical nanopore geometry; and surface charges that exert electrostatic interactions with mobile ion charge carriers. For silica or aluminum oxides, surface charges are negative due to the deprotonation of terminal oxide groups when solution pH is higher than surface pK_a_, making cations the main charge carriers. The polarity may switch to neutral or even positive at lower/acidic pHs upon the protonation of available functional groups.

AAOs are known to have highly uniform cylindrical nanochannels across the membrane, often densely packed in a hexagonal organization. The nanochannels are open on one side of the membrane, referred to as oxide/electrolyte side (O/E), and can contain a porous oxide layer that is permeable but as a barrier for IT at the metal/oxide (M/O) interface on the opposite end, as sketched in Scheme 1B. AAO structures have been tuned successfully in nanochannel diameter, channel density or interchannel spacing, and channel length or membrane thickness by adopting different acids, temperature, and other synthetic conditions.^1^ While the types of space charges within the barrier oxide layer (BOL) are generally accepted to arise from 1, oxygen and/or aluminum vacancies; and 2, impurities such as the acid anions used during synthesis, their inhomogeneous distributions across the BOL are riddled with conflicting results likely a consequence of the dynamics of the intrinsic space charges of BOL exchanging with environment under operando conditions.^10,41−46^ The BOL causes asymmetry across the AAO membrane, which has been shown to induce steady-state ICR. AAOs are chosen in this report to study the IT dynamics in macroscopic ensemble membranes and also to achieve those selective IT dynamics properties with the consideration of their high nanochannel/nanopore density and easy accessibility. A postsynthesis ion-exchange strategy is established to overcome the uncertainties associated with the BOL space charges in the as-synthesized AAOs.

## Results and Discussion

### IT Hysteresis in the Rectified *I*–*V* Curves

**Figure 1:**
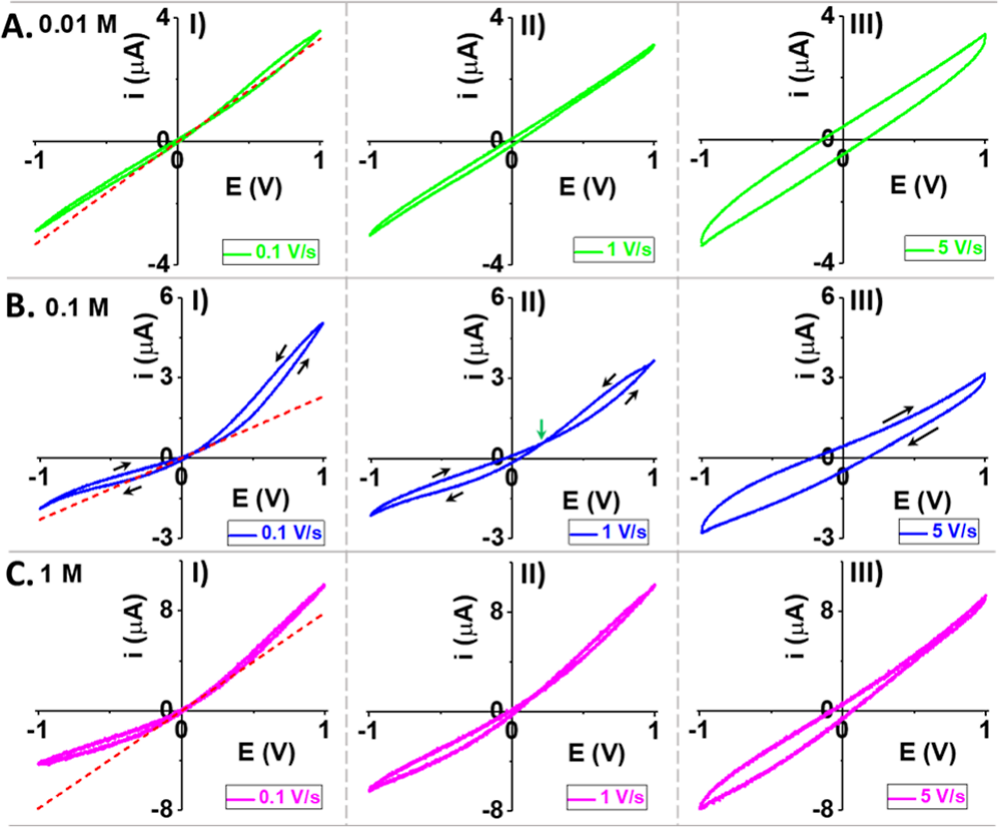
Electrokinetic ionic current features at different KCl concentrations (A) 0.01 M, (B) 0.1 M, and (C) 1 M and scan rates (I) 0.1 V/s, (II) 1 V/s, and (III) 5 V/s. Data recorded with an AAO membrane after ca. 23 h post fabrication. The dotted redlines in 0.1 V/s panels are calculated from the forward scan current at +0.1 V and across the origin (0, 0) to illustrate the departure from linear ohmic behavior. Black arrows in 0.1 M, panel B, show potential scan directions with forward defined as from negative to positive potential scans (the same trend for other concentrations). Green arrow in (B) (II) points to the position of the cross-point.

Free-standing AAO membranes are obtained after the M/O interface of the BOL is exposed upon chemical etching of the aluminum substrate. The process is associated with drastic increases in the cross-membrane ionic conductance and optical transparency. Chronoamperometric techniques are employed to monitor and control the etching together with in situ optical imaging. Representative results are shown in Figure S1 along with synthetic details. Schemes S1 and S2 and inserted optical images show the custom-built devices for synthesis and measurements. The AAO membranes display characteristic ICR and memristor-type hysteresis features in a wide range of ionic strength and scan rates. The trends are illustrated in Figure 1, supported by additional ion concentrations and scan rates, in Figure S2. Under triangular waveform in cyclic voltammetry with the applied potential scanned back and forth, both forward and backward current branches display nonohmic ICR behavior and cross at a potential away from the origin (0, 0). Two obvious hysteresis *I–V* loops in the LC and HC states are separated by this cross-point. The conductivity “memorizes” the previous conductivity state in that at a given potential such as +0.6 V in 0.1 M 1 V/s panel, the current amplitude is lower when the system is shifting from lower to higher conductivity states (potential scan from negative to positive, ground in the O/E chamber), compared to the current in the reversal scan from HC to LC.

Observation of the hysteresis and ICR at high ionic concentrations (1 M in Figure 1, 2 M data in Figure S2) is significant to emphasize because those phenomena arise from electrostatic effects which are known to be suppressed at high ionic strength in earlier single nanopore studies where surface charges play determinant roles.^47^

Hysteresis and ICR are more prominent at intermediate ionic strength such as 0.1 M than lower or higher ionic concentration, e.g., 10 mM and 1 M. With the increase in the scan rate at a given ion concentration, the HC current and HC loop decrease, while LC current and LC loop increase due to less time for ion concentration polarization and thus weaker ICR.^48,49^ The cross-point does not shift at intermediate scan rates when the current arises primarily from IT across the nanochannels. When the scan rate is sufficiently high, the cross-point shifts to more positive and ultimately disappears (5 V/s here) when the negative RC loop (resistance-capacitance) in HC states is completely overshadowed by the normal capacitance charging current, which arises from the electrical double layer (EDL) of the AAO membrane exterior surfaces.^38^ The *I–V* hysteresis loop should not be confused with the (dis)charging of classic EDL, which is qualitatively consistent with the RC loop in the 5 V/s panels (0.01 and 0.1 M) featuring parallel current branches corresponding to a normal capacitance phase shift with respect to the applied electrical field. Instead, the HC RC loop has an opposite phase shift compared to normal capacitance, as apparent inductance, due to the intrinsic electric field being in the same direction with the applied field.^11,50,51^ Of note, the transport component with a positive phase shift remains, attested by the current gap in HC being distinguishably smaller compared to the LC (nonparallel current branches). At slower scan rates, a simple ICR curve is observed, i.e., the two current branches overlap. This steady-state response is expected because the delays in the redistribution of ions would be insignificant with slow changes in the potential stimulus.

### Accessibility to Multiple Excitatory and Inhibitory Conductivity States

**Figure 2:**
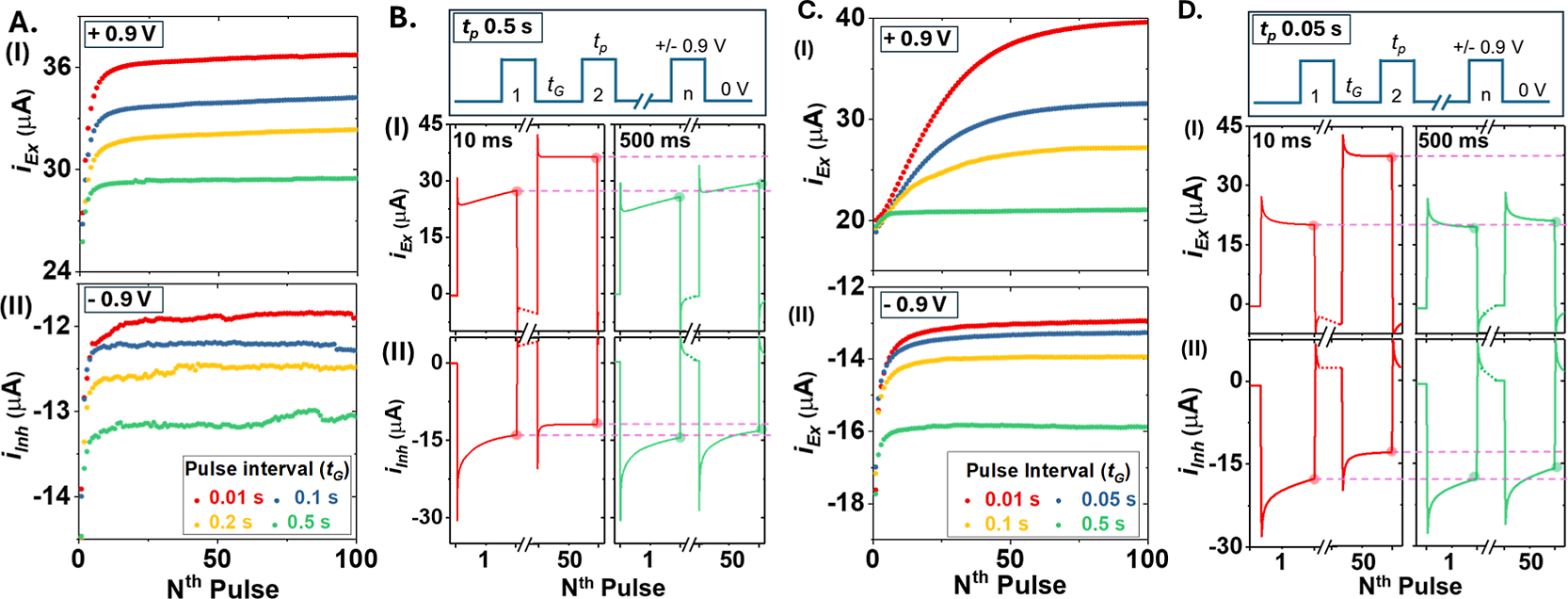
Excitatory (I, top, + 0.9 V) and inhibitory (II, bottom, −0.9 V) conductance features under pulse train stimulus. The pulse duration is 0.5 s in (A,B) and 0.05 s in (C,D), while the potential is 0.0 V during pulse intervals/gaps (*t*_G_). The current is recorded at 1 ms per point in 0.1 M KCl. Pulse #1 and #50 with intervals of 10 and 500 ms are enlarged, respectively, in (B,D) to illustrate current changes in individual pulses. The trend over repeated pulses is shown in (A,C) by plotting the last points in each pulse against pulse number. The current during pulse intervals at 0.0 V is approximately zero and omitted for clarity.

The current–time traces in Figure 2 highlight short-term memory effects and the capability to access multiple conductivity states by simply controlling the potential waveform. Under a potential pulse to either enrich or expel mobile ions, different excitatory and inhibitory states, i.e., increased and decreased conductance upon repeated stimulation, respectively, are stimulated by adjusting the pulse interval or duration (and obviously magnitude, not shown), while keeping the others constant. Within each pulse regardless of pulse polarity, a sharp current decrease can be seen immediately after the potential steps due to the classic RC discharging (RC stands for the time constant of the electrochemical cell, resistance-capacitance equivalent model), i.e., characteristic potential step *I–t* exponential decay profiles. After this initial decay, the current increases gradually and over repeated pulses under a +0.9 V stimulus, as shown in panel B. (I) A longer pulse causes faster increases (panel A vs C, first few cycles). Further, a higher plateau in current amplitude is reached with shorter pulse intervals (10 ms vs 500 ms, first and 50th pulses in panel B/D) despite with the same accumulation time under a constant pulse period. With zero voltage applied during pulse interval, i.e., no stimulus building on from the previously enriched/depleted states directly illustrates short-term memory effects.^52^ Otherwise, the current in later pulses would be the same compared to the earlier ones without an increase or decrease.

Similar trends are observed in LC states, where stronger ion depletion is reached with either a shorter pulse interval or longer pulse duration or both. The plateau inhibitory states become slightly less stable only under long pulse duration and long intervals, likely due to the leaching of impurity ions explained next, which can be overcome through adopting high-purity materials and synthetic optimization or simply avoiding extreme concentration polarizations. Obviously, many different states can be accessed readily and consistently by applying different potential waveforms (potential amplitude, duration, and interval) and in different ionic strengths following the same principle. Overall, these properties illustrate the reversibility and consistency of accessing different conductance states.

### Structural Characterization by Scanning Electron Microscopy

**Figure 3:**
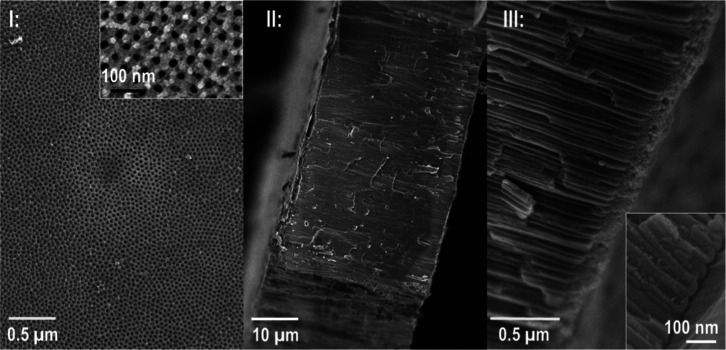
SEM characterization of the AAO membranes. I. Top view of the O/E surface. The inset shows the enlarged view of individual nanochannel opening. II. Side views of the whole AAO membrane. III. Tilted view of the M/O surface with BOL (enlarged as inset). Scale bars vary in different panels.

As shown in Figure 3, the average diameter of the nanochannels is measured to be 25 ± 3 nm from the opening on the O/E surface, and the nanochannel length or membrane thickness is 38 ± 1 μm. The amorphous BOL at the end of the M/O interface is better seen in the inset of panel III with a thickness of 20 ± 1 nm. Additional scanning electron microscopy (SEM) images and statistical analysis data are provided in Figure S3 and Table S1. These structures are largely consistent with those in AAO membrane literature,^53^ despite industrial grade aluminum (99% purity) being used here to demonstrate the robustness of our understanding applicable to low-cost easily accessible materials. The nanochannel density is about 3.4 ± 0.3 × 10^8^ per mm^2^ nanochannels based on the average interchannel spacing of 40 ± 7 nm. In other words, the rectified and hysteretic selective IT properties are observed from an impressive ensemble of nanochannels, 15% of the total mm^2^ sized membrane collectively.

The AAO nanostructures suggest that BOL is the only symmetry-breaking entity across the AAO membranes. Indeed, complete removal of the BOL via chemical etching eliminates the ICR and hysteresis to generate linear ohmic *I–V* curves (Figure S4). Loss in transport selectivity is expected due to the highly symmetric cylindrical geometry of those nanochannels functioning as the transport limiting region. In reference to the rich literature where the type, density, and distribution of space charges in AAO BOL depend on synthetic conditions and may evolve over time,^42−45^ we attribute the transport selectivity to space charges rather than the geometric curvature of the BOL. The hypothesis is confirmed by the characterizations and simulation discussed next, and two additional observations in 1, both ICR and hysteresis from the as-synthesized AAOs intensify over the first few days and then decrease (Figure S5); and 2, occasionally, some of the as-synthesized AAO membranes display HC states and negative phase shift with opposite polarity in the negative potential polarity (ground/reference on the O/E side, Figure S6). Noteworthily, the alignment of the BOL/membrane with the bias polarity remains unchanged in both cases. Accordingly, a postsynthesis ion-exchange strategy is developed to tune the polarity of the ICR and hysteresis and better control the transport selectivity.

### Postsynthesis Control on the Polarity of *I*–*V* Rectification and Hysteresis via Ion Exchange

**Figure 4:**
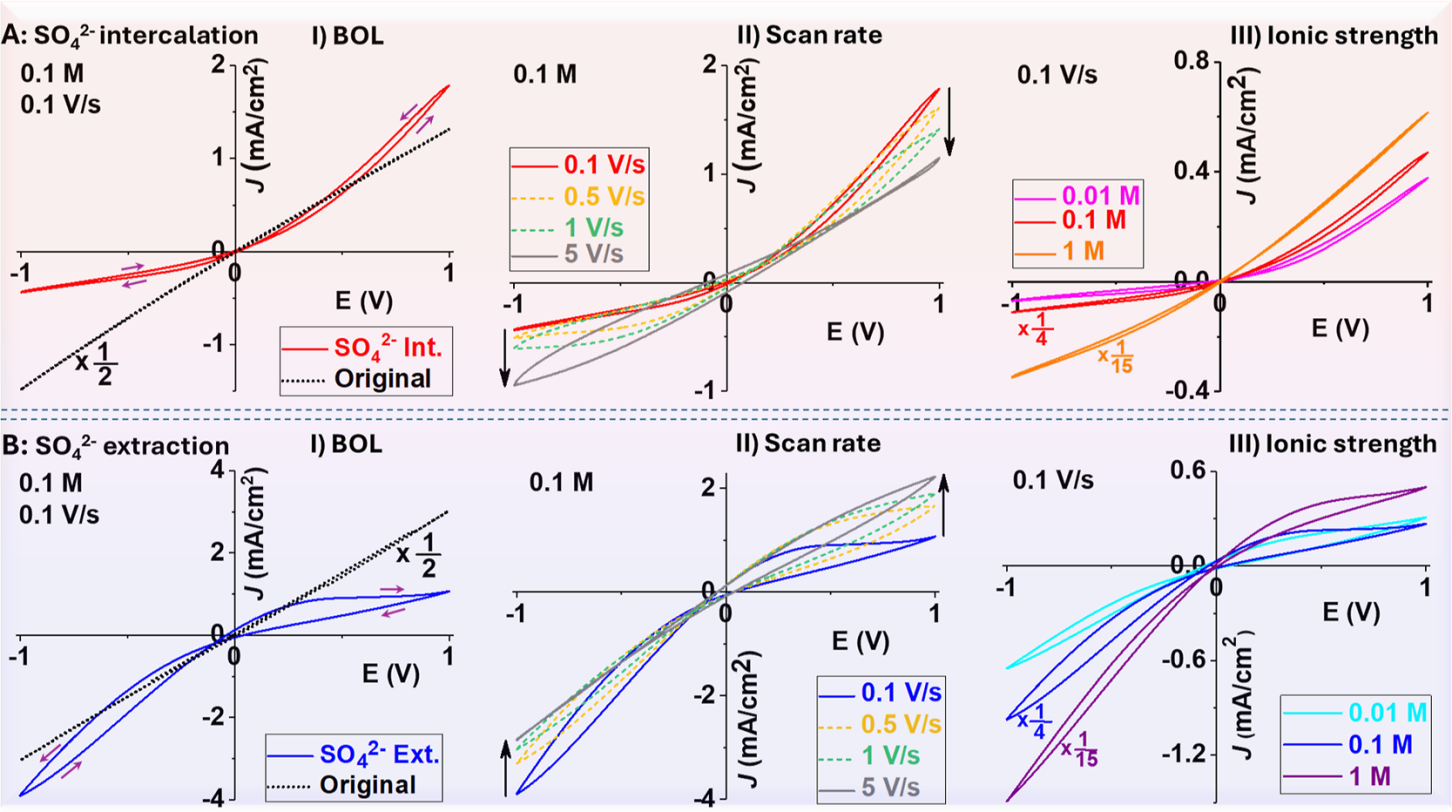
Inversion of *I–V* curves of AAO membranes upon (A) sulfate intercalation and (B) extraction. Enhanced ICR and hysteresis are shown from the KCl ionic current before and after ion exchange (I) and further at different scan rates (II) and KCl concentrations (III). The factors listed next to some current curves were applied for direct visualization of the rectification and hysteresis. Current density is based on the AAO area estimated from corresponding optical images illustrated in Figure S1E.

Generally speaking, space charges in BOL arise from oxygen and aluminum defects as well as impurities such as the acid anions used in the synthesis. In our syntheses with sulfuric acids in the O/E chamber, SO_4_^2–^ anions would be embedded in the BOL, with concentration decreases from O/E toward M/O in principle.^1^ However, as well-documented in the literature and in our experience, uncertainty remains to accurately control the space charge distribution especially combined with the dynamic IT behaviors after the exposure of the M/O interface. Multivalent ion exchanges facilitated by an applied electrical field are found to be highly effective to tune the polarity of the ICR and hysteresis consistently. As shown in Figure 4, electrolysis of the as-synthesized AAOs in the presence of excess SO_4_^2–^ in solution under a constant bias (−0.9 V) overnight is found to induce strong positive ICR (A) (I–III). Conversely, negative ICR and hysteresis (B) (I–III) are induced by electrolysis in a 1:1 electrolyte without SO_4_^2–^. These much stronger ICR and hysteresis properties display the same dependence on ionic strength and scan rate as the as-synthesized AAOs explained in Figure 1. *I*–*V* data from additional membranes displaying similar trends are provided in Figure S7. To correlate the polarity of the ICR and hysteretic *I–V* loops, a straightforward way of thinking is that the enrichment in the hysteretic charges and positive phase shift (negative capacitance) in the *I–V* loop always occur at the HC states of ICR regardless of the potential bias definition or ion exchange-induced inversion.

The intercalation or extraction of the SO_4_^2–^ ions appears to be irreversible as the polarity of the ICR and hysteresis can no longer be reversed under comparable conditions after the original electrolysis. This is postulated to structural transformation of the porous oxides associated with the exchanges of multivalent anions characterized next. Meanwhile, effectiveness in purifying the electrolyte ions after synthesis and ion exchanges, as well as switching ionic solutions between measurements is crucial to obtain reproducible dynamic transport features. In between those operations, the AAO membranes are routinely tested in DI water over multiple cycles of potential scans until an insignificant current is obtained (Figure S8). Further, preliminary stability test results are provided in Figure S9, in which insignificant current drift over consecutive potential scans is observed as well as retained ICR and hysteresis loops over weeks/months. Overall, the postsynthesis ion exchange establishes consistent ionic memristor features, either in positive or negative polarity, at high current density in addition to high ionic strength (1 M) in macroscopic membranes. As always, the results, including the control experiments, are reproduced with different batches of samples (generally in the range of 5–10 or more for each ICR-type AAO).

### X-ray Photoelectron Spectroscopy Characterization of the Space Charges and Their Distribution across BOL

**Figure 5:**
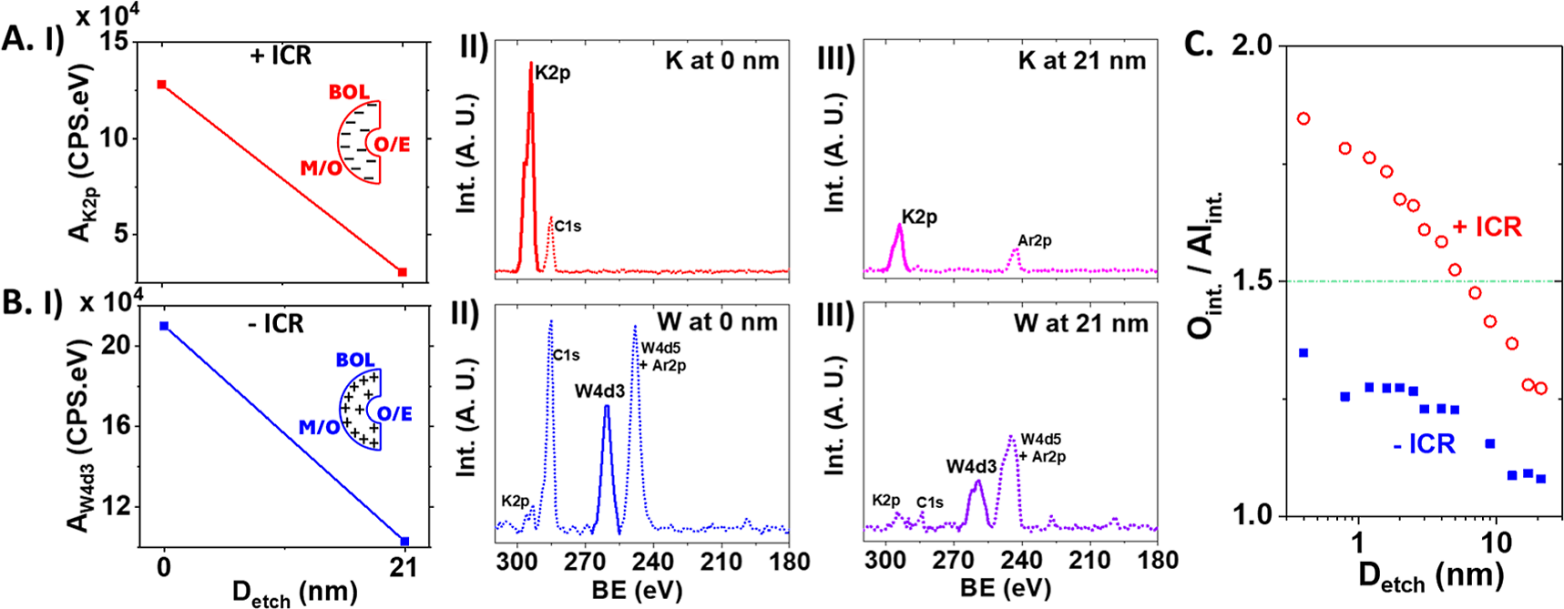
XPS analysis of AAOs with (A) + ICR and (B) –ICR before and after stepwise etching and (C) O/Al intensity ratio. Etching depth *D*_etch_ at 0 nm corresponds to the M/O surface, while 21 nm is approaching the end of the BOL on the O/E side. For direct comparison of these signals in (A) and (B): the data at M/O and O/E of BOL in (I) and spectrum panels (II) and (III) have the same intensity scale (in arbitrary units). All signals, K, W, O, and Al, were normalized by their respective sensitivity factors in quantitative comparisons. Baseline treatments and detailed data analysis in the Supporting Information. Additional peaks with inconsistent intensities arise from carbon impurities and residual Ar from chamber purging.

The polarity and distribution of space charges across BOL are characterized by XPS depth profiles upon stepwise etching in Figure 5. Since the AAO membranes are tested in KCl solutions and thoroughly cleaned prior to XPS analysis (efficacy confirmed by recording conductivity in nanopure water, Figure S8), the signals from excess electrolyte ions represent net counterions required for electroneutrality and thus reveal the space charge polarity and density. For AAOs with + ICR, K^+^ is detected with an intensity significantly higher at the M/O surface. Anion signals are much weaker or absent. From the –ICR AAOs, K^+^ is marginally observed above the baseline. Strong anion tungstate bands are consistently observed with higher intensity at the M/O interface. The tungstate were generated from the W electrode during the electrolysis under +0.9 V which were then embedded in BOL compensating the space charges.^1−3^ Based on the excess K 2p and W 4d3 signals versus the respective counterions and at different locations, + ICR is correlated with higher negative space charges at the M/O surface that decreases toward the O/E side, while –ICR has higher positive space charges and is similarly higher at the M/O surface. Full survey spectra before and after stepwise ion beam lithography etching of the BOL from these same AAOs are provided in Figure S10, and results displaying similar trends from additional AAOs are shown in Figure S11.

The efficacy of the SO_4_^2–^ intercalation is confirmed from the depth profiles of the SO_4_^2–^ intensity (peak at 170 eV) in Figure S12.^54−56^ Near the M/O interface where ion exchange starts, the SO_4_^2–^ intensity is stronger after the intercalation treatment from AAOs displaying + ICR. The SO_4_^2–^ signal is much weaker at the M/O interface from –ICR samples after extraction treatments. This is attributed to the replacement by tungstate during the extraction electrolysis. Toward the O/E end, the SO_4_^2–^ intensity decreases but not zero which reflects the anion impurity from the original synthesis assuming insignificant SO_4_^2–^ redistribution within BOL. The trend is consistent from multiple membranes but should only be treated qualitatively or semiquantitative due to the low signal intensity.

Detailed XPS depth profiles in Figure 5C provide further insights on the space charge distributions in BOL. An overall ratio of O/Al at 3:2 = 1.5 corresponds to a neutral composition of Al_2_O_3_. Above and below 1.5 would indicate Al vacancy and O vacancy, or negative and positive space charges, respectively. This is under the assumption that acid anion impurities are insignificant compared to the presence of O- or Al-vacancies. With the oxygen abundance overestimated by sulfate or tungstate (WO_4_^2–^) in the –ICR samples, the nearly constant O/Al within the first few nanometers of the M/O interface suggests higher positive permanent space charges at the M/O interface. The depth analysis on + ICR AAOs, consistent with the excess K^+^ determined from survey scans, further reveals higher negative space charges at M/O that decreases continuously to neutral within the first few nanometers and then positive space charges toward O/E dominated by Al vacancies.^1,42−45^

### Mechanistic Insights from Finite Element Simulations

**Figure 6:**
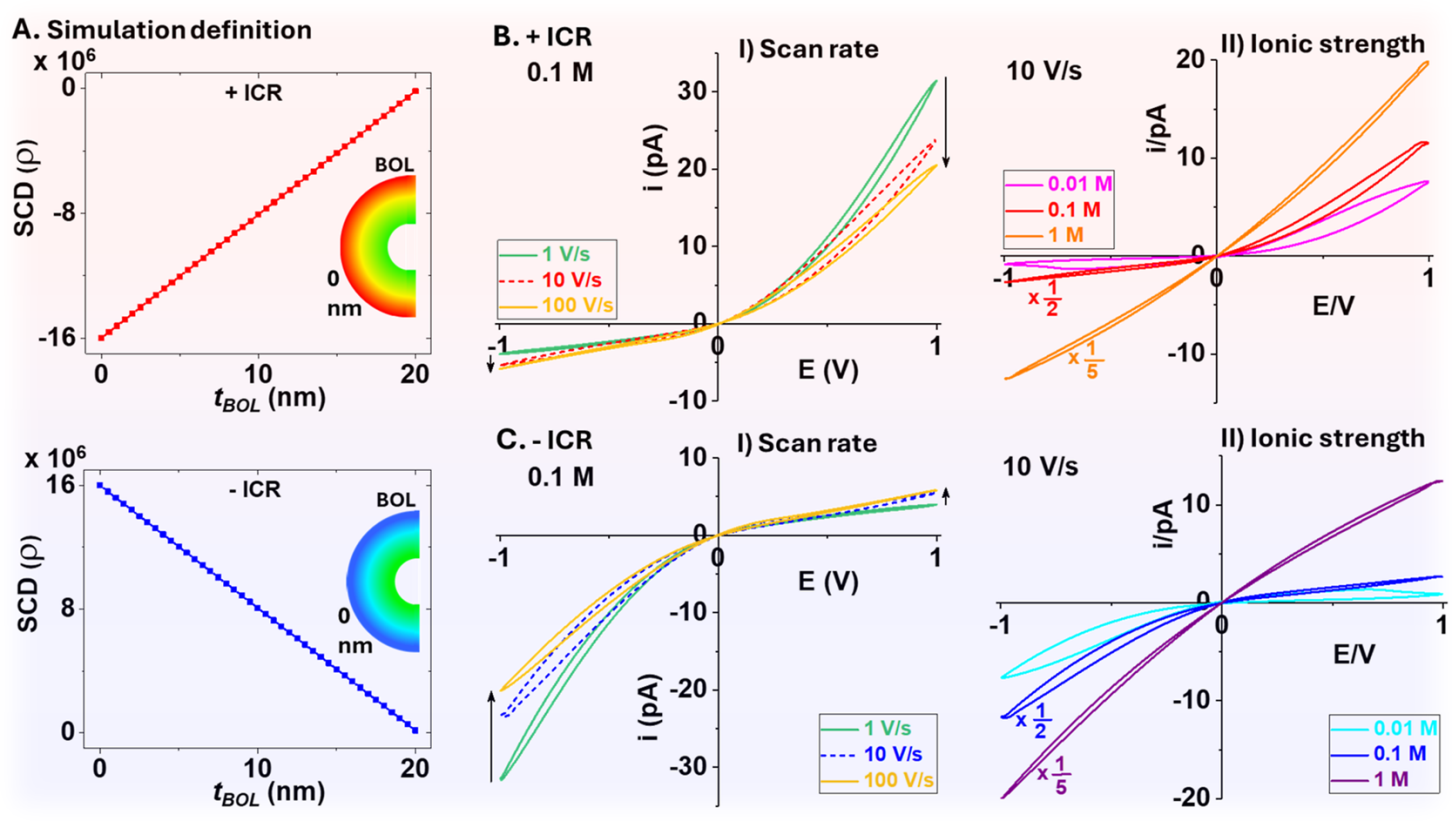
SCD gradients on memristor polarity. (A) High negative (top) and high positive (bottom) space change density that decreases nearly linearly from M/O (0 nm) interface across the BOL. (B) (I) and (C) (I) scan rate effects under constant ionic strength and (B) (II) and (C) (II) ionic strength effects at constant scan rate on ICR and hysteresis. The 0.1 and 1 M curves were normalized by the listed factors for visual comparison within the same current scale.

The negative and positive space charges and their gradual changes in density across the BOL are essential differences between the positive and negative AAO memristors. For simplicity to elucidate mechanistic understanding, a linear gradient in space charge density (SCD) is defined via eq 1, plotted in Figure 6A with negative (top) and positive (bottom) polarities. The continuous gradient across the hemispherical BOL is based on our experimental characterization and those in literature.^10^
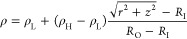
1

The origin of the (*r*, *z*) integration coordinates is at the center of the hemispherical BOL. ρ_H_ and ρ_L_ are high and low space charges at the outside M/O (*R*_O_) and inside (*R*_I_) interfaces, respectively. An axial symmetry along nanochannel centerline (*r* = 0) is used in solving coupled Poisson, Nernst–Planck, and Navier–Stokes equations (Comsol). The AAO nanochannel and BOL structures are defined based on SEM characterizations. ρ_H_ is defined at 1.60 × 10^7^ C/m^3^ (0.1 e/nm^3^), 100 times higher than ρ_L_, values within the normal range in literature.^10^ Noteworthily, variations in those materials parameter values would not affect the observed trends. An important factor that affects or dominates IT is the correction factor used for diffusion coefficients in BOL. This is determined experimentally from the conductivity differences before and after BOL removal (about 5 times increase in current, see in Table S2, data from measurements illustrated in Figure S4). Additional simulation details are described in the Supporting Information, including structure schemes and boundary definitions in Figures S13 and S14.

The positive ICR and hysteretic features at different scan rates and ionic strengths in panel B, and negative ICR ones in panel C, clearly correlate with the negative and positive SCD gradients. The dependences of the ICR and hysteretic loops with scan rate and ionic strength reproduce the experimental trends in Figures 1 and 4 successfully. As controls in Figure S15, the *I–V* curves are symmetric under opposite potentials with constant SCD definitions across the BOL, regardless of positive or negative. Much weaker hysteresis loops and departure from linearity in *I–V* curves can be resolved, which likely result from the BOL curvature to be explained in a follow-up report.

**Figure 7:**
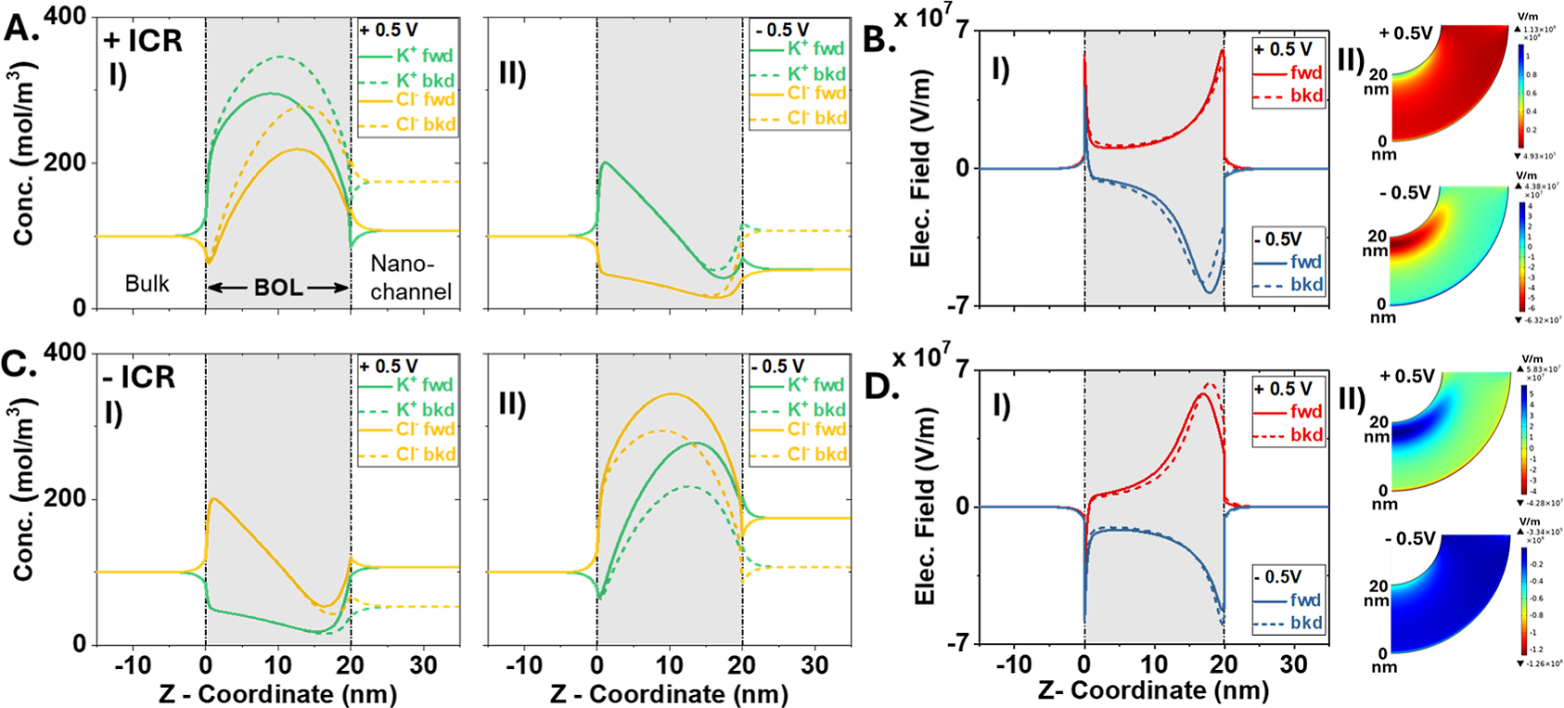
Concentration profiles (AC) and electric field distribution (BD) across BOL with + ICR (AB) and –ICR (CD). Reference/ground electrode is in the right chamber facing the nanochannels. Data acquired in 0.1 M KCl at 10 V/s along centerline (*r* = 0). *Z* = 0 nm represents the M/O interface, and *Z* > 21 nm is inside nanochannels. The same *y*-axis scale is used for direction comparison. Solid curves are forward (fwd) scan (from −*V* to +*V*) and dotted curves from backward (bkd) scan (from +*V* to −*V*). Note 2D surface plots of the electrical field are provided in BD (II) where the *z*-direction is vertical.

The concentration profiles and electrical field distributions in Figure 7 reveal the charge carriers and transport limiting regions inaccessible from experiments directly. With high negative space charges at M/O in the + ICR scenario, K^+^ is enriched under positive bias in HC states. The concentration of co-ions Cl^–^ near M/O is lower than bulk due to electrostatic repulsion by the space charges but enriched further inside the BOL due to the significantly enriched K^+^ ions to maintain electroneutrality. Both K^+^ and Cl^–^ are enriched more in the backward scans (to LC) as the system “memorizes” the previous HC states. This is more obvious inside nanochannels where the KCl concentration is significantly higher than that in the bulk. Panel B reveals that the potential drops almost exclusively across BOL and establishes a high electrical field localized near both interfaces of BOL, i.e., the limiting region for IT.

In LC states (-0.5 V panels), Cl^–^ is expelled significantly to be lower than bulk, especially at the O/E interface where the K^+^ concentration is also lower than bulk. The electrical field is predominantly localized near the interface facing the nanochannel. These observations lead to an intriguing conclusion that the transport-limiting region is shifted primarily near the nanochannel interface, and K^+^ is more dominant as charge carriers. In the backward scan, both counterions and co-ions are still higher in concentration than forward scan, though the absolute concentrations are lower as it takes longer time to reach LC from HC potentials. The KCl concentration in the nanochannel is also lower than +0.5 V but remains closer to the bulk. An interesting and counterintuitive feature is the positive electrical field under −0.5 V near the M/O interface. By plotting the differences in K^+^ and Cl^–^ concentrations and an alignment in the concentration gradient with the electrical field in Figure S16, the opposite field is attributed to the dynamic concentration polarization effects at the sharp M/O boundary.

Similarly, as shown in panels C and D, positive space charges make Cl^–^ main charge carriers in –ICR results, in particular LC states, where all features are opposite in polarity.

The gradient of space charges in the BOL is fundamentally different from surface charges in nanopores or other nanodevices, but both establish an intrinsic electrical field exerting similar impacts on IT. When the intrinsic electrical field is in the same direction with the applied field, the HC state is established. An opposite cancellation effect determines the LC states. Regardless of the applied potential magnitude, increasing toward higher *V*_HC_ (or *V*_LC_) or decreasing while scanned back toward the *V*_CP_, where *V*_HC_, *V*_LC_, and *V*_CP_ refer to the potentials at HC/LC states and cross-point, respectively, the intrinsic electrical field continues to enrich mobile ions over time in HC states and deplete in LC states. Accordingly, the backward current amplitude (from *V*_HC_ to *V*_LC_) is higher over the forward branch [*V*_LC_, *V*_HC_] at the same potentials because the previous states of the backward scan have more charge carriers over the forward ones, forming the *I–V* hysteresis HC/LC loops. The potential at the cross-point is a direct measure of and compensates for the intrinsic electrical field across the BOL. A negative phase shift in capacitance is observed in HC because the intrinsic electrical field assists the applied field to drive IT current and a normal capacitive phase shift in LC loop as the two fields are in opposite directions.^16,57^ It is worth emphasizing that molecular–level interactions, such as ion pairing and (de)solvation, are not captured in the continuum theory adopted in this study. Future works are needed to gain fundamental insights for generalizable and predictive capabilities.^37,58^

## Conclusions

To summarize, characteristic memristor behaviors in pinched current–potential loops are resolved in BOL-retained AAO membranes. Rectified ionic current forms two *I–V* hysteretic loops by crossing at a point away from the origin (0, 0) driven under a cyclic triangular potential waveform. Positive and negative polarities in the hysteresis and rectification directions are achieved by the extraction or intercalation of multivalent anions (SO_4_^2–^). XPS analysis reveals high negative (or positive) space charges at the M/O interface that decrease across BOL in AAOs displaying positive (or negative) hysteresis and rectification. Accordingly, these emerging phenomena are attributed to the gradients of space charges in the BOL. Finite element simulation further confirms the effects of space charge gradients and reveal spatiotemporal insights on the IT. Main charge carriers and enriched charges are determined to be cations in +ICR and anions in −ICR. Both experiments and simulation observe strong ICR and hysteresis at high ionic strength 1–2 M electrolytes and over a large range of potential scan rates or dynamic range. Short-term memory effects are demonstrated under pulse train stimulus. It is worth mentioning that the polarity, preferred transport direction, is determined by the relative direction of the gradients of space charges with respect to the bias polarity, neither the geometric nanostructures nor the absolute bias definitions and surface charge. Exciting potentials in energy, separation, iontronics, and other applications are envisions supported by the efficacy over a wide range of ionic strength and operation conditions, as well as great tolerance in structural variations in ensembles and low-cost materials.

## Supporting Information Available

Synthesis, experimental and simulation details; SEM image and analysis; XPS and additional current–potential data from other AAO membranes (PDF)
