# Supplementary material for: Anodized Aluminum Oxide Membrane Ionic Memristors
Source: J Am Chem Soc. 2025 Mar 20;147(13):11089–97. doi: 10.1021/jacs.4c16835 (PMC11969543; doi:10.1021/jacs.4c16835)
Supplement: Supplementary file 1 — ja4c16835_si_001.pdf [file ja4c16835_si_001.pdf]

Supporting Information

Anodized Aluminum Oxide Membrane Ionic Memristors

Dipak Baram,‡ Maksim Kvetny,‡ Sarah Ake, Ruoyu Yang, Gangli Wang*

Department of Chemistry, Georgia State University, Atlanta GA 30302

Table of Content

Material and Methods

Finite Element Simulation

Two-step Anodization, Chemical Milling and Anion Exchange

List of Schemes, Tables and Figures:

Scheme S1. Anodization setup and two-step anodization procedure.
Scheme S2. In-house made H-cell holder for AAO membrane measurements.
Figure S1. Electrochemical milling for the removal of aluminum substrate under optical monitoring.
Figure S2. ICR and hysteresis in 0.05 M to 2 M KCl under 0.1 V/s (black) and 0.5 V/s (red) scan rates.
Figure S3. Representative SEM images from different AAO membranes and pore size measurements.
Table S1. SEM data summary.
Figure S4. BOL removal by chemical etching monitored by conductivity changes.
Figure S5. Evolution over time of the positive rectification and hysteresis of as-synthesized AAO membranes.
Figure S6. AAO membrane displaying negative ICR and hysteresis.
Figure S7. Effects of sulfate intercalation and extraction from another AAO membranes demonstrating the reproducibility of trends in Figure 4.
Figure S8. Electrolyte removal confirmed by conductivity decrease in DI water.
Figure S9. Short- and long- term stability tests of AAOs after ion exchanges.
Figure S10. XPS survey scan of BOL surface of AAO.
Figure S11. Additional XPS survey scan analysis of counterions for space charges in AAO membranes with + ICR and – ICR respectively.
Figure S12. High resolution XPS spectra of sulfur bands across BOL.
Table S2. Correction factor for diffusion coefficient in BOL determined from experimental 0.1 M KCl current at +1 V (i_{HC}) and -1 V (i_{LC}) with and without BOL.
Figure S13. Schematics of the 2D axisymmetric geometry and space charge density definitions.
Figure S14. Adaptive mesh element definition (A) and boundary conditions (B).
Figure S15. Simulated *I-V* curves with constant space charge density (SCD) across BOL (A) negative and (B) positive.
Figure S16: Electric field, concentration difference (Δ conc.) and its gradient ($\frac{dC}{dz}$).

Materials and Methods

Materials

All materials and reagents are used as received. For anodized aluminum oxide (AAO) membrane synthesis and holder fabrication: aluminum 1100 plate, 1/16” (Metal Supermarket, 1100 grade purity 99 %), FLGPCL01 Resin (Clear, Formlabs), ePTFE gasket sheet, 1/16" (VSP Technologies). Reagents for AAO fabrication: 70.00 % $HClO_4$ (Sigma Aldrich), 200 Proof Ethanol, USP (Decon Laboratories), 95.00 % H_2SO_4 (Sigma Aldrich), 85.00 % H_3PO_4 (Fisher Scientific), ≤ 99.00 % CrO_3 (Alfa Aesar), 36.50 % HCl (Fisher Scientific). For measurements: ≥ 99.00 % KCl (Sigma Aldrich), 99.90 % Ag wire 0.50 mm (Sigma Aldrich), 99.90 % Pt foil 0.025 mm and Pt wire 0.50 mm (Themo Scientific). For Ag wire coating: 97 % $FeCl_3$ (Sigma Aldrich) and for SO_4^{2-} intercalation: ≥ 99.00 % K_2SO_4 (Sigma Aldrich), 99.95 % Tungsten wire 0.50 mm (Alfa Aesar).

Instruments and Measurements

Electrochemical data were acquired using Gamry Reference 600 (Gamry Co.) inside a Faraday cage at room temperature. Two spiral Ag/AgCl wires (dia. 0.5 mm) were used as reference electrode (RE) and working electrode (WE), with Pt. foil as counter electrode (CE). Data collection rate was 1 mV per point in cyclic voltammogram and 1 ms in the pulse train measurements. The potential range is limited within ± 1 V to avoid possible water splitting. The Ag/AgCl wires were activated in saturated $FeCl_3$ solution regularly to ensure constant potential (insignificant drift due to the redox current). In addition, a separate Pt wire as a working electrode and a Ag/AgCl wire as working sense (4-electrode setup) were used to confirm the consistency in the measured current-potential curves with the 3-electrode setup. Unless otherwise specified, all results are repeated with different synthetic batches of AAOs. Multiple I-V curves (more than 3 cycles, generally 5-10 cycles) were recorded in each measurement. The late cycles overlap so one complete cycle is plotted for clarity. The first segment or cycle is discarded because the initial solution condition is uncontrolled (which is a common practice in voltametric measurements).

For electropolishing and anodization: DC power supply (BK Precision 9130), ceramic Thermoelectric Module (0.15" thick,15.4 V, 83.9 W at D122 °F, McMaster-Carr), Haake K20 Circulating Chiller C1 (500 W, Marshall Scientific), Digital overhead stirrer (SCILOGEX SCI20-S, 50-2200rpm). For removal of excess aluminum: Standard Infuse/Withdraw Syringe Pumps (PHD 2000, Harvard Apparatus). For AAO membrane holder: 3D printer (Form 2, Formlabs), photopolymer resin (Formlabs, FLGPCL01, Clear).

Electrolyte removal and replacement

After fabrication, between measurements in different ionic concentrations, and before XPS data collection, AAOs were thoroughly cleaned using DI water (18.2 MW) under scanning potential (+/- 1V) at 0.1 V/s scan rate until the current decreased 100 times (99 %) compared to 10 mM KCl current (less than 100 nA).

X-ray Photoelectron spectroscopy (XPS) analysis

AAO membranes were heated at 70 °C for about 42 h and kept in vacuum chamber at 30 torr for 20 h prior to XPS analysis. The chemical composition of the BOL was analyzed with Thermo K-Alpha XPS System (Thermo Fisher Scientific) with a monochromatic Al Kα source (KE = 1486.6 eV), a 180° double focusing hemispherical analyzer, and a 128-channel detector. The base pressure was below 2.5 $x10^{-7}$ mbar. The X-ray beam diameter was set to 300 µm with raster size 1.5 x 3 mm^2, and a charge neutralizer (flood gun) was used to compensate for undesirable surface charging effect with slow electrons and Ar^+ ions. To measure the depth profile of AAOs, an Ar^+ ion beam with 500 eV to 3000 eV energy operating at a beam current of 2 to 5.47 mA was used. The sputtering rate of the ion beam was based on raster size, beam current, beam energy and angle factor in reference to those from Ta_2O_5. The sputtering time in the depth profile was

calibrated with the sputter rate. The high-resolution elemental spectra was obtained with pass energy 50 eV with step size 0.1 eV in depth profiling; and 200 eV with 1 eV step size in survey scans.

For the high-resolution depth profile data analysis, XPS peak fitting was performed after Shirley type background correction and Gaussian-Lorentzian fitting using Avantage Surface Chemical Analysis software (version 5.9925, Thermo Fisher Scientific). The peak intensity (area) was normalized by the sensitivity factor of the corresponding element to determine the elemental ratios. Analysis of potassium and tungstate from the survey scan was performed by considering the linear type of background in the Origin (2017).

SEM Characterization:

AAO membranes were characterized with the Hitachi SU8230 FE-SEM (Hitachi High-Technologies Corporation) with cold cathode field emission (CFE) gun for improved imaging and analytical performance. Image j was used for nanopore dimension determination.

Finite Element Simulation

The ion transport dynamics through AAOs were studied by finite element Modeling using the COMSOL Multiphysics v. 5.5, Electrochemistry module. Three interfaces of Transport of Diluted Species, Electrostatics and Laminar Flow, and corresponding equations Nernst-Planck (NP), Poisson and Navier-Stokes (NS) were utilized to perform simulation. Multiphysics coupling was enabled to solve Nernst-Planck-Poisson (PNP) equation and *Poisson-Navier-Stokes (PNS) equation.* Additionally, 'Convection' was enabled within the *Transport of Diluted Species to couple* electroosmotic flow (EOF) in the transport dynamics.

Table 1. Relevant equations solved by software for each of the employed interfaces

Nernst-Planck (NP) Equation	Poisson (P) Equation	Navier-Stokes (NS) Equation
$J_j = -D_j \nabla C_j - \frac{z_j F}{RT} D_j C_j \nabla \varphi + C_j v$	$\rho_E = -\varepsilon\varepsilon_0 \frac{\delta^2 \phi}{\delta y^2}$	$d_s \frac{dv}{dt} = -\nabla P + \eta_s \nabla^2 v + f$

The NP equation describes three types of contributions to mass transfer processes. The first term corresponds to the diffusional flux, the second term with migrational flux and the third term to convective flux (e.g. electroosmotic flow, EOF). Poisson's equation correlates the charges (density) with electrostatic potential. Finally, essentially the stating of Newton's First Law (F = ma) for a fluid, the NS equation consists of three terms each correlated with a type of force: pressure force (first term), frictional force (second term) and field force (third term).

The finalized 2D, axisymmetric geometry is constructed based on experimental results shown in Fig. S13-14. Some boundaries underwent additional segmentation as this enabled us to increase the mesh resolution within areas of interest (i.e. transport limiting regions). Adaptive mesh with customized sized was used. Near the boundaries highlighted in blue in Figure S14 A., a maximum mesh size of 0.1 nm was imposed. 'Free Triangular' mesh was applied to the remainder of the geometry further away from transport-limiting region to reduce computational cost.

Two-step Anodization, Chemical Milling and Anion Exchange

AAO fabrication

Illustrated in Scheme S1, the reactor consists of an anodization head (Scheme S1, panel I) and three separate temperature-controlled solution baths: for electropolishing, oxide removal, and for anodization steps. The anodization head consists of two ¼ inch copper plates, acting as the electrodes, electrically isolated from one another by a 20 mm ePTFE insulating block. Each copper electrode has a threaded hole to allow the aluminum electrodes to be attached. To maintain the proper temperature of the aluminum samples, a thermoelectric module coupled with a water supply chamber (heat exchanger) was attached to the anode. Continues water supply at hot side of thermoelectric module enhances its cooling efficiency on another side (cold side where reaction head is attached). Both anodization steps use a common, 1.1 L H_2SO_4 bath with an overhead stirrer to provide vigorous stirring. The bath temperature was maintained at 0°C using a recirculating chiller with a Haake K20 Circulating Chiller C1 (500 W chilling capacity) connected via a water jacket. A DC power supply was used for both electropolishing and anodization.

Anodized aluminum oxide membranes were fabricated following literature procedures with modifications[2]. The 1/16" Aluminum 1100 plate (Metal Supermarket) was first sectioned into 6 mm x 40 mm rectangular sections. The aluminum sections were then rinsed with acetone followed by ethanol and annealed in a furnace at 450 °C for 4 hours before being allowed to cool to room temperature inside the furnace over the next 12 hours. **Step 1:** Individual aluminum sections were then electropolished with 15 % $HClO_4$ in ethanol, at a constant current density of 400 mA/cm^2 until a mirror finish was achieved, temperature is maintained at ~5 °C using dry ice/ethanol bath and the thermoelectric module with the continuous stirring of solution. **Step 2:** The first anodization step was performed at 25 V in a 20 % H_2SO_4 solution held at 0 °C for 15 minutes, with the maximum current density capped at 800 mA/cm^2 and 25 V DC potential. Active thermoelectric module cooling was maintained until the current density dropped below 100 mA/cm^2, (or typically for 60 seconds). **Step 3:** The resulting oxide layer was then stripped with a solution of 3% H_2CrO_4 in 5% H_3PO_4 at 60 °C over the course of 5 minutes under vigorous stirring via a magnetic stirring element. **Step 4:** The final anodization step was performed similar to step 2 conditions but for 45 minutes instead of 15 minutes. The resulting anodized aluminum sections were then thoroughly washed with deionized water to remove any excess acid and proceed for milling process.

Chemical milling setup and procedure

Both the milling (to remove the aluminum substrate) and conductivity measurements were performed with a membrane holder, consisting of three separate parts; a central support plate sandwiched between two solution reservoirs. The membrane is secured between the support plate and one of the reservoirs with an ePTFE gasket, giving access to the membrane surface for the opening step (removal of aluminum) and allowing the additional reservoir solution to be added for conductivity experiments without compromising the integrity of the seal. A detailed diagram is provided in Scheme S2 and milling set up at Figure S1 AB.

The anodization process creates an AAO layer on both sides of the aluminum section. One side is sacrificed to fabricate a free- standing AAO membrane after the removal of aluminum backing. Briefly, a small section of aluminum was exposed by piercing one side of the electropolished surface with a sharp needle. This area is then exposed to a concentrated HCl (10% v/V) solution that etches away the aluminum overtime (the oxide layer on top collapse alongside, see in Figure S1 B). Fresh HCl solution is continuously infused at a rate of 0.25 mL/min with a syringe pump and the resulting waste and excess solution is continuously removed via suction (building vacuum line). The entire etching process is monitored over time by measuring the current under a constant potential applied across the two reservoirs. Throughout the etching process the current rises steadily followed by an abrupt increase once sufficient aluminum has been removed to expose a free-standing oxide membrane. Longer etching time increases the exposed area and correspondingly the transport current. An example current profile is provided in Figure S1 C. Additionally, due to the greater optical transparency of AAO over aluminum metal, a blue LED backlight was used to

both confirm the removal of aluminum material and to estimate the area of AAO exposed. Etching of the newly exposed AAO barrier oxide layer (BOL) at M/O interface is possible during the HCl exposure process but this is deemed insignificant during these procedures, because the dissolution rate of oxide is much slower in the commonly used HCl removal of aluminum from AAO membranes. The argument is further supported by: 1. SEM images of the BOL taken after shorter and longer exposure times having identical features, 2. Transport features being qualitatively consonant regardless of exposure time variations.

Intercalation and Sulfate exchange at Barrier Oxide Layer (BOL) of AAO membrane

Ion exchanges are performed with an AAO sandwiched in the H-cell holder. The sulfate intercalation was performed by two ways. 1. The chamber facing M/O is filled with 0.5 mL of 0.1 M K_2SO_4 and the other chamber with 0.5 mL of 1 mM KCl. Two tungsten electrodes were inserted with the one in M/O chamber as working and the one in O/E reservoir as ground. A constant potential of -0.9V was applied for 24 h using function generator. 2. M/O filled with 0.5 mL of 0.1 M K_2SO_4 with empty other chamber and leave for 24 h at ambient condition. For extraction/exchange, both chambers are filled with 0.5 mL 1 mM KCl and a constant potential of + 0.9V is applied for 24 h.

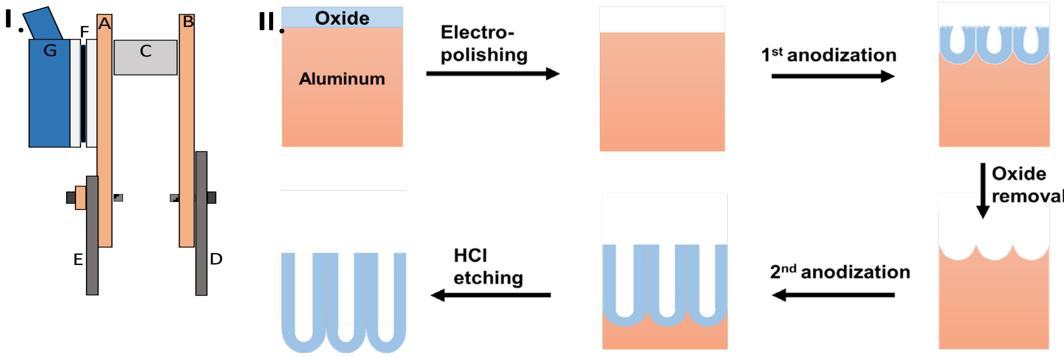

Scheme S1. Anodization setup and two-step anodization procedure.
I. Anodization head diagram: **A.** Copper Anode base, **B.** Copper Cathode base, **C.** ePTFE insulator, **D.** Aluminum Cathode, **E.** Aluminum Anode (being anodized), **F.** Thermoelectric module, **G.** Thermoelectric module heat exchanger. **II.** Two-step anodization synthetic procedure. Not drawn to scale.

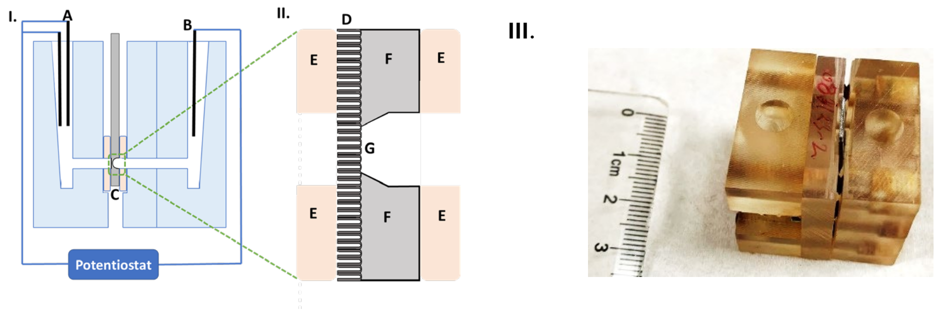

Scheme S2. In-house made H-cell holder for AAO membrane measurements.
Panel **I**: **A.** electrolyte reservoir with Ag/AgCl electrode (RE) and Pt foil (CE) inserted. This reference/ground electrode faces the cylindrical nanochannels with the O/E interface at the bottom. **B.** electrolyte reservoir with Ag/AgCl working electrode inserted, facing M/O interface after Al removal. **C.** AAO membrane portion exposed to electrolyte solution enlarged in Panel **II**: with E represent ePTFE

gaskets providing the tight seal of the AAO membrane between the two electrolyte solutions. **F** is remaining Aluminum metal after chemical etching intact with AAO nanopores **D**. **G** is exposed portion of membrane to the electrolyte solution after etching. **III.** Real AAO membrane holder, AAO membrane with Al plate sandwiched in between two compartments with reservoirs (right and left wholes).

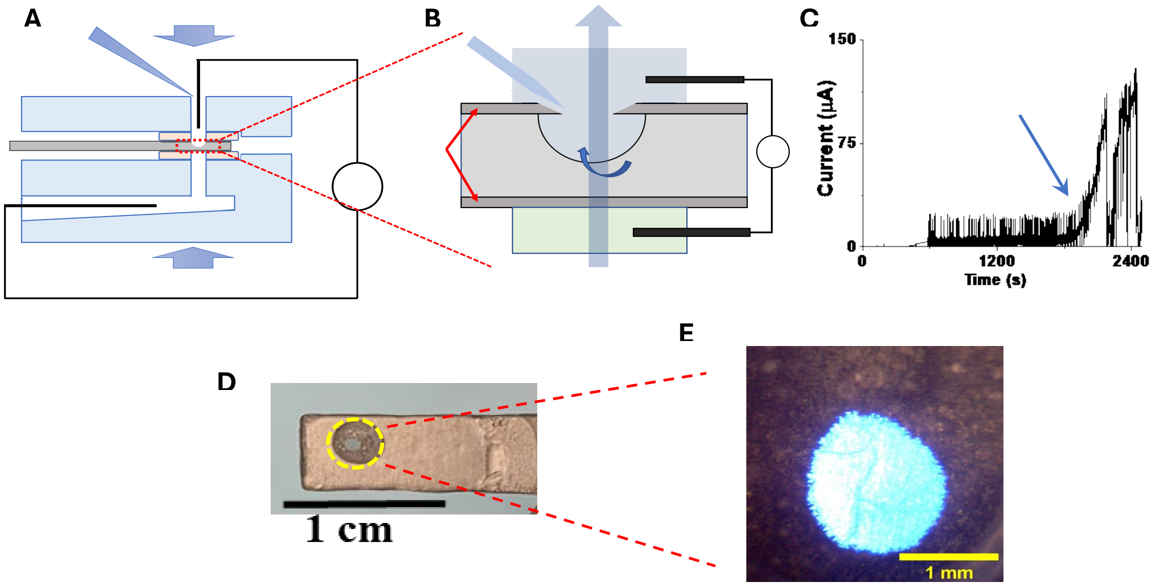

Figure S1. Electrochemical milling for the removal of aluminum substrate under optical monitoring. **A**. Milling sketch of Al using 10% HCl, **B**. Magnified portion of Al (being removed) intact with AAO. The side to be sacrificed is pierced with a needle to expose a section of aluminum and HCl is pumped into the exposed area reacting with the aluminum and creating a cavity, monitoring of current between the two reservoirs is also started. Once the aluminum is removed and a free-standing membrane is exposed the current increases sharply. **C**. I-t plot of the milling process, the arrow indicates the transition point in the current when the aluminum is fully removed, and a free-standing membrane is achieved. **D**. Optical image of the original membrane (in yellow circle) after removal of excess aluminum. Semitransparent AAO is surrounded with grey color oxidized aluminum. **E.** An optical image of the exposed membrane backlit by a blue LED light positioned just below the semitransparent AAO holder. optical image of the exposed membrane backlit by a blue LED light positioned just below the semitransparent AAO holder.

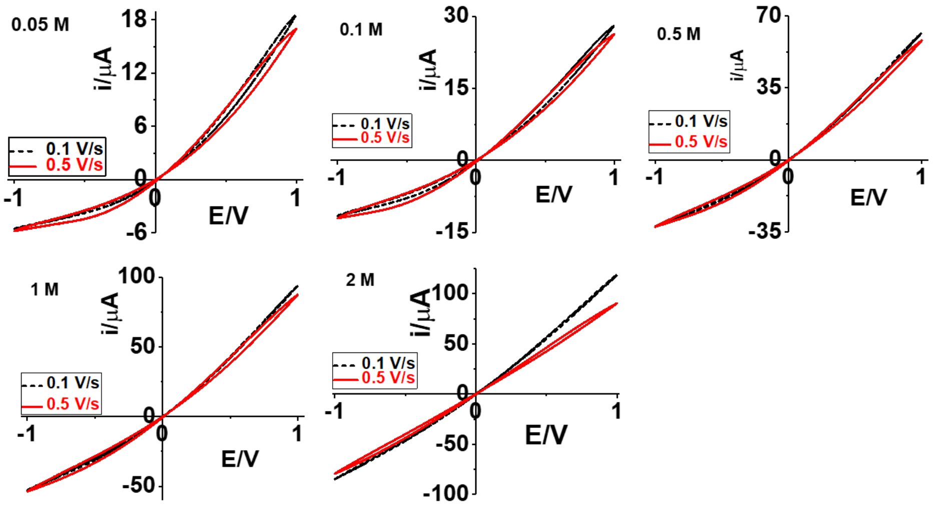

Figure S2. ICR and hysteresis in 0.05 M to 2 M KCl under 0.1 V/s (black) and 0.5 V/s (red) scan rates. Data from another AAO membrane to demonstrate the reproducibility of trends in Figure 1.

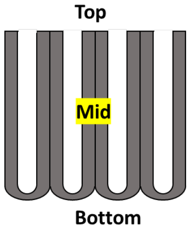

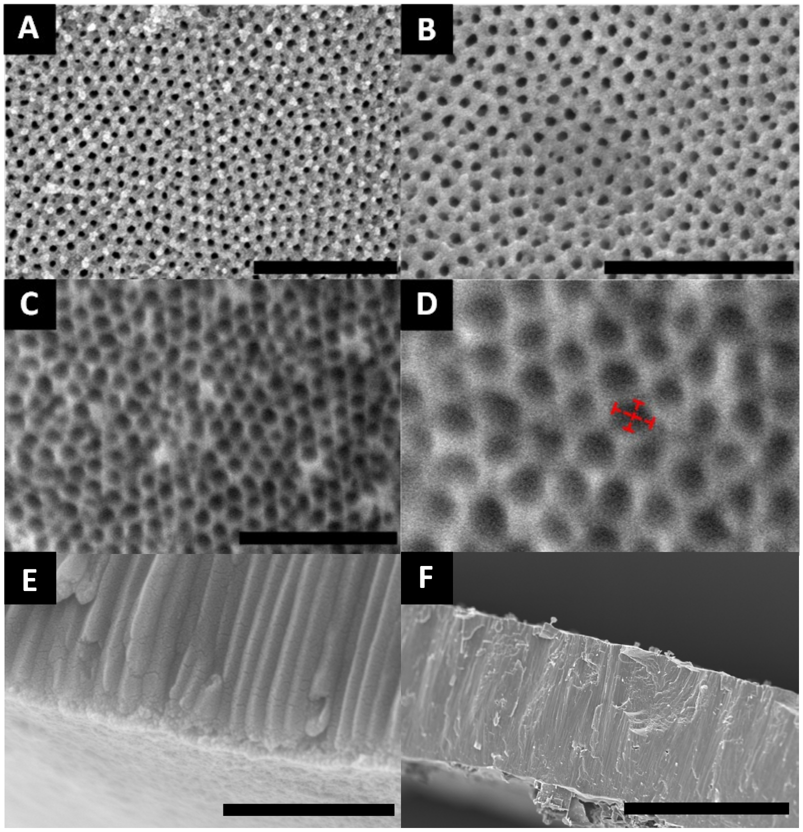

Figure S3. Representative SEM images from different AAO membranes and pore size measurements. **A-C** are top views from O/E side. Red marks in **D** illustrate a direct measurement of pore sizes. Due to pore asymmetry, each measured pore size is the average of the long axis and the perpendicular of axis. **E**. Magnified side-view of the metal oxide interface after aluminum removal, broken sections used to estimate BOL thickness. **F**. Cross section view of oxide membrane used to determine overall membrane thickness. Scale bars in **A-E** are 500 nm and 40 mm in **F**.

Table S1. SEM data summary.

**AAO**	**Measurement position**	**OD (nm)**	**ID (nm)**	**BOL thickness (nm)**	**Membrane thickness (mm)**	**Pore density (per mm^2)**
**AAO1**	Top	62	25	21	37.26	3.67 x 10^8
	Middle	70	25	19	-	
	Bottom	65	28	18	-	
**AAO2**	Top	59	28	-	36.82	3.66 x 10^8
	Middle	66	26	20	-	
	Bottom	68	28	20	-	

**AAO3**	-	-	22	-	39.15	3.05 x 10^8
**AAO4**	-	-	20	-	-	3.39 x 10^8
	**Average**	**65**	**25.3**	**19.6**	**37.7**	**3.44 x 10^8**
	**SD**	**3.65**	**2.77**	**1.02**	**1.01**	**2.53 x 10^7**

Measurement results from four AAO membranes are provided. In area-based analyses, Image J was used to determine the areas of individual pores. The statistical mean and standard deviation in diameter was calculated by assuming circular pore cross section. The nanochannel length or membrane thickness was determined from the images with the whole membrane cross sections.

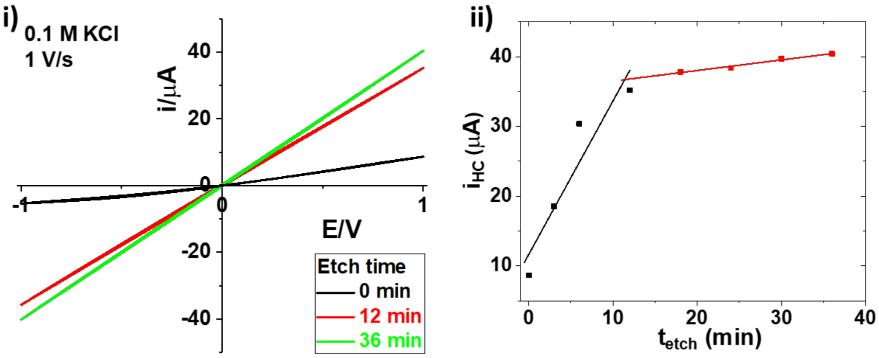

Figure S4: BOL removal by chemical etching monitored by conductivity changes.
I-V curves were collected in 0.1 M KCl about every three minutes after chemical etching in a mixture of 1.25 % (v/V) H_3PO_4 and 0.75 % (w/V) H_2CrO_4 followed by DI water washes. The original AAO membrane display ion current rectification and hysteresis (black curve in i). The current (conductivity) increases with etching time and those rectified features disappear completely (showing no ICR hysteresis) after about 12 min.

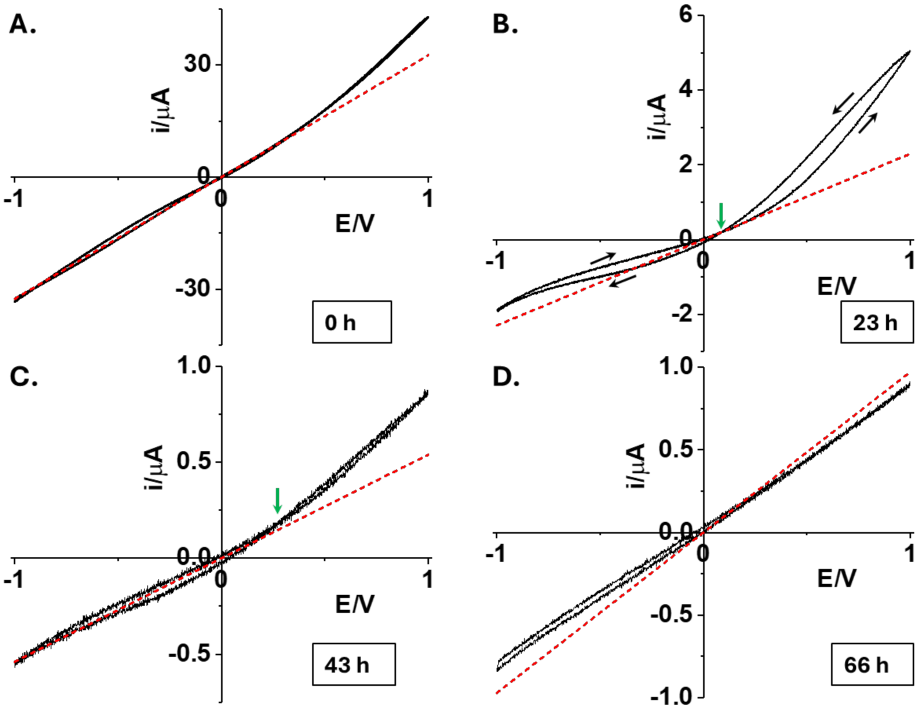

Figure S5. Evolution over time of the positive rectification and hysteresis of as-synthesized AAO membranes.

Data recorded in 100 mM KCl at 100 mV/s scan rate. The red line is calculated from the current at + 0.1 V and across the origin (0, 0) to illustrate an ohmic behavior. Green arrows in B and C indicate the position of the cross point. Black arrows in panel B indicate potential scan directions to highlight the current hysteresis loops. The current amplitude is lower in the forward branch which is defines as potential scans from lower to higher conductivity (negative to positive potential), compared to the backward branch at the same potentials. The count of hours is the time after membrane fabrication. Note the current scale decreases from panel A to D.

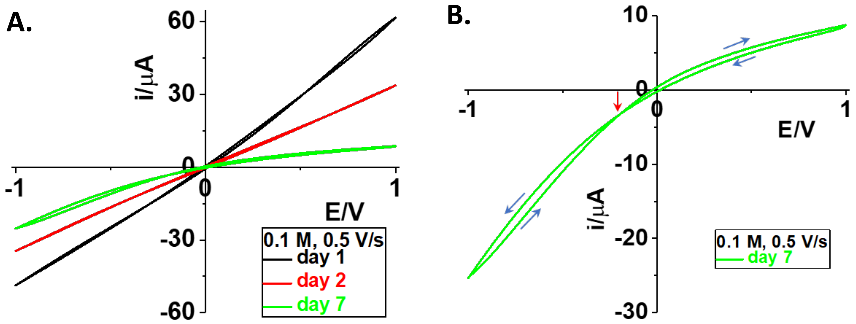

Figure S6. AAO membrane displaying negative ICR and hysteresis.

A. *I-V* data at 100 mM KCl solution over several days from the same AAO membrane and **B.** magnified *I-V* data of day 7. Monotone arrows indicate scan direction for a given segment of the corresponding color. Red arrows indicate the position of Crosspoint.

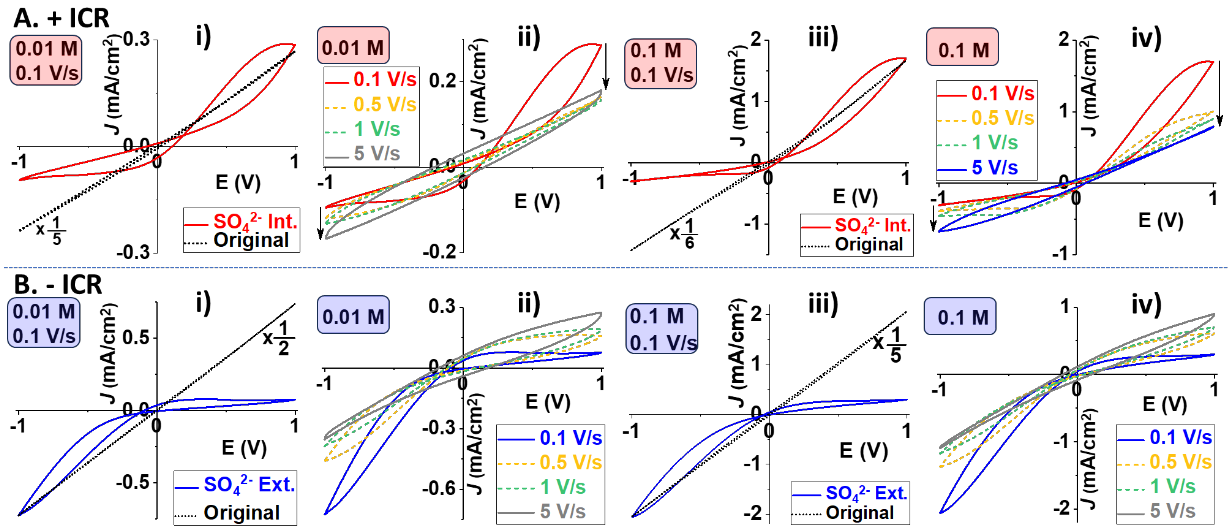

Figure S7. Effects of sulfate intercalation and extraction from another AAO membranes demonstrating the reproducibility of trends in Figure 4.

A. + ICR and **B.** – ICR. Panels i) and iii) compare the original and after ion exchange in 0.01 M and 0.1 M KCl respectively. Panels ii) and iv) show scan rate dependence in the two concentrations.

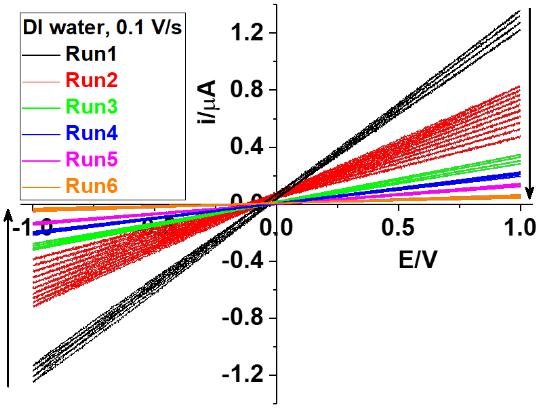

Figure S8. Electrolyte removal confirmed by conductivity decrease in DI water.
Each run contains 4 to 10 Cycles, until the current in later cycles becomes less than 1 % of the current signal in the 10 mM KCl solution.

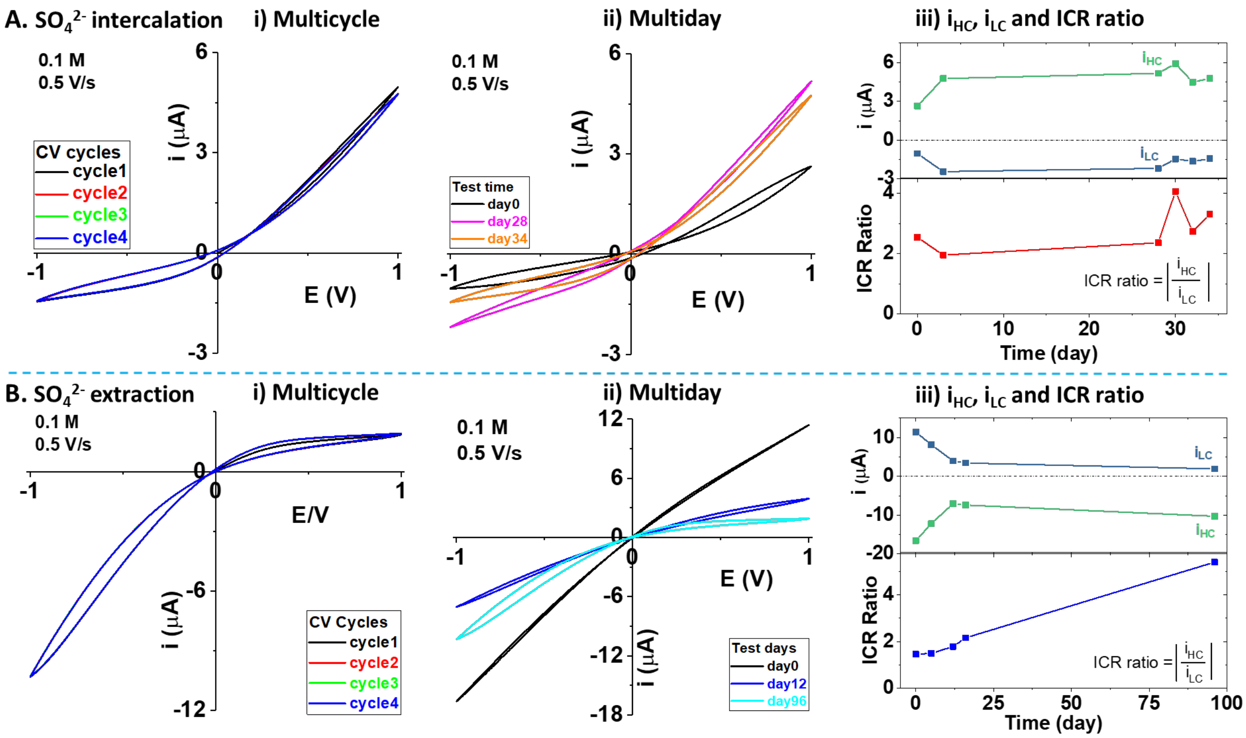

Figure S9. Short- and long- term stability tests of AAOs after ion exchanges. Current values in panels iii) were from ±1 V at the respective HC and LC states. Consecutive potential scan cycles show negligible drifts (panels i), which is generally the case with the AAOs soaked in solution over hours (not shown). The absolute current may drift after days/weeks but the ICR and hysteresis features remain prominent (panels ii). Note: practical factors such as the storage conditions were not controlled. The results are provided for qualitative trends rather than for quantitation purposes.

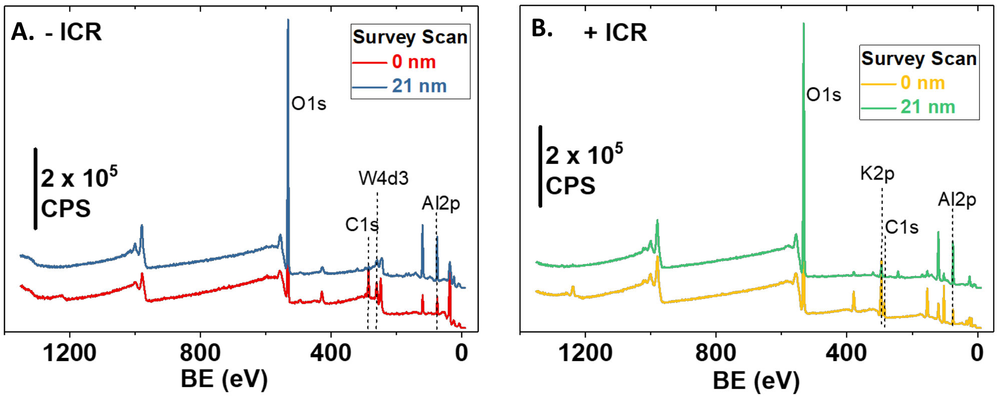

Figure S10. XPS survey scan of BOL surface of AAO.
A. - ICR AAO and **B.** + ICR AAO at the M/O interface, 0 nm and at O/E interface (21 nm). Intensity in counts per second (CPS) vs binding energy (BE)

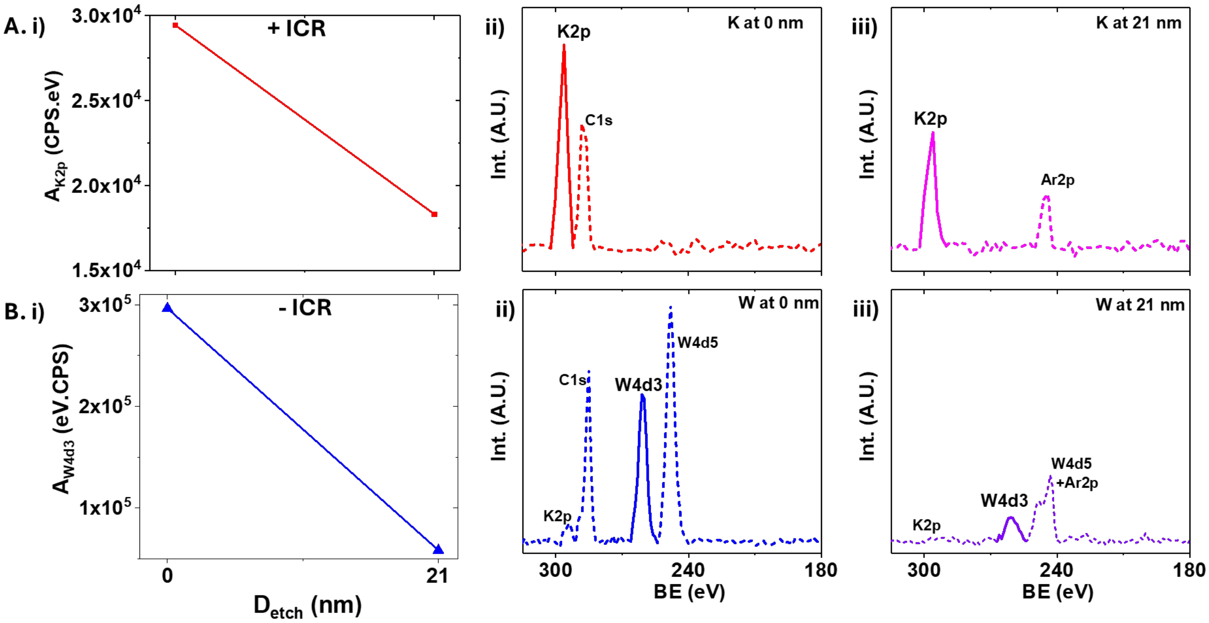

Figure S11. Additional XPS survey scan analysis of counterions for space charges in AAO membranes with + ICR and – ICR respectively.
A. Excess potassium and **B.** Tungstate. The area under curve of K2p (A_{K2p}) and W4d3 (A_{W4d3}) from another AAO were plotted to demonstrate the consistent trends in Figure5.

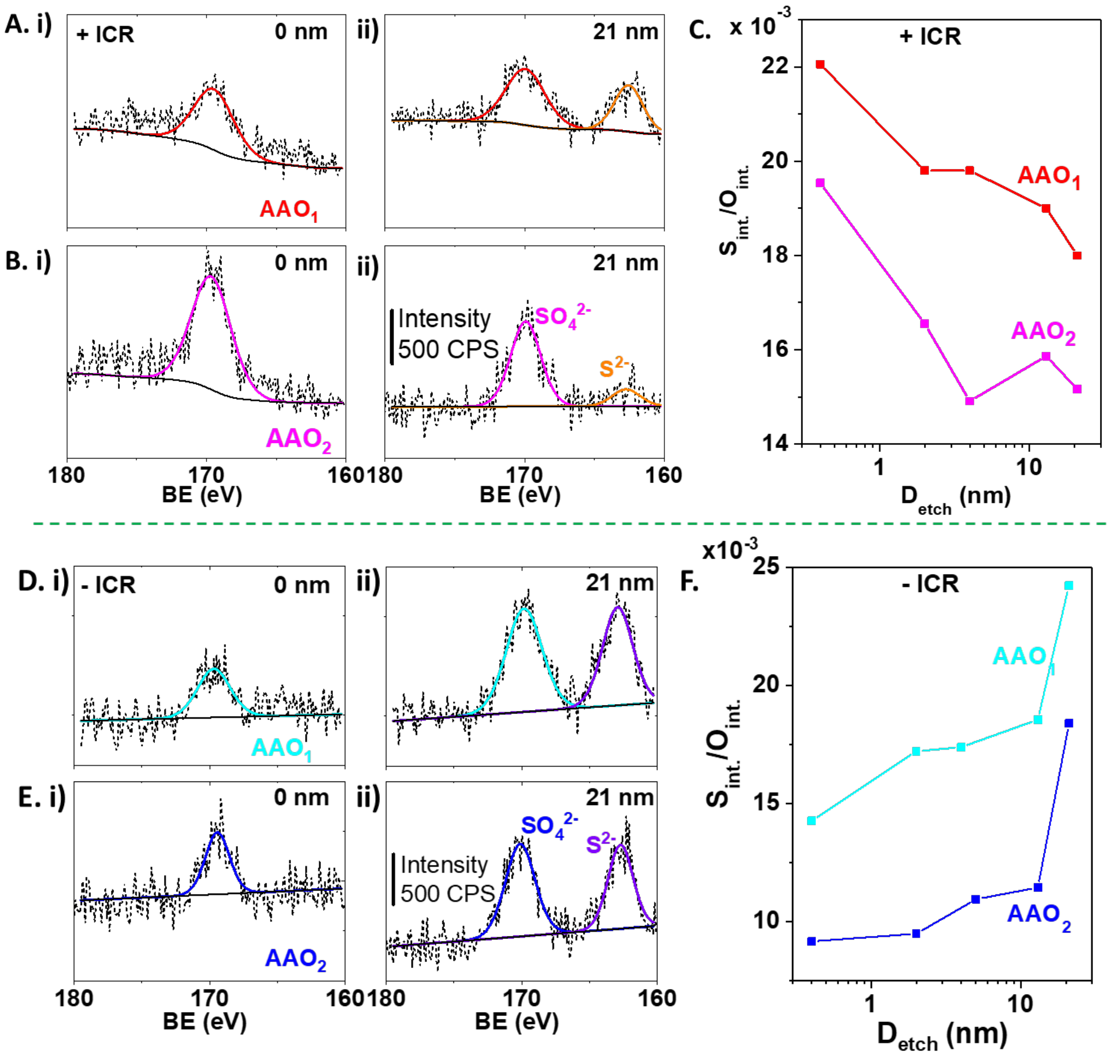

Figure S12. High resolution XPS spectra of sulfur bands across BOL.
Upper panel **AB.** two + ICR AAOs and lower panel **DE**. two – ICR AAOs at i) 0 nm (M/O) and ii) 21 nm (O/E) along with trend of S intensities (peak at ~ 170 eV) across BOL for + ICR AAOs **C.** and – ICR AAOs **F.** Peak at around 170 eV assigned to SO_4^{2-} and around at 162.5 eV assigned to sulfide (S^{2-})[3-5]. At each point, the peak area of SO_4^{2-} was normalized by peak area of oxygen in the same spectrum and corrected with corresponding elements sensitivity factors.

Table S2. Correction factor for diffusion coefficient in BOL determined from experimental 0.1 M KCl current at +1 V (i_{HC}) and -1 V (i_{LC}) with and without BOL.

AAOs	(BOL)i_{LC} (mA)	(NoBOL)i_{LC} (mA)	$\frac{(NoBOL)i_{LC}}{(BOL)i_{LC}}$	(BOL) i_{HC} (mA)	(NoBOL)i_{HC} (mA)	$\frac{(NoBOL)i_{HC}}{(BOL)i_{HC}}$	Average
**AAO_1**	-39.39	-209.70	5.32	38.72	210.75	5.44	5.38
**AAO_2**	-11.49	-79.42	6.91	15.23	80.74	5.30	6.10
**AAO_3**	-8.15	-40.19	4.93	12.98	40.39	3.11	4.02
**AAO_4**	-84.81	-311.57	3.67	91.45	313.76	3.43	3.55
**Average**			5.21			4.32	**4.76**

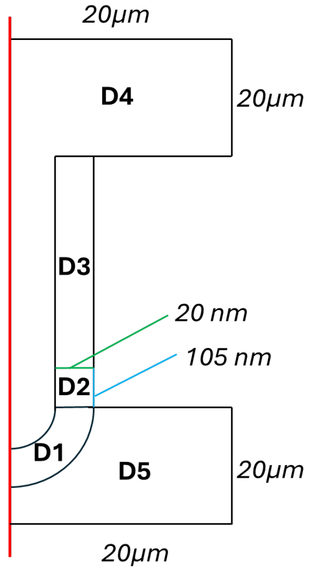

D1 = BOL with space charge density $\rho = \rho_L + (\rho_H - \rho_L)\frac{\sqrt{r^2+z^2} - R_I}{R_0 - R_I}$

D2 = AAO wall – adjacent to BOL with $\rho = \rho_L + \frac{\left(\rho_H - \frac{\rho_H - \rho_L}{3R_O}(z)\right) - \rho_L}{R_O - R_I}(r - R_I)$

D3 = AAO wall – bulk with $\rho_{bulk} = \rho_L$

D4 &D5 = Electrolyte filled nanochannel and reservoirs with *r* = FC*(zK*cK+zCl*cCl)

The origin of (r, z) integration coordinates is at the center of the hemispherical BOL. ρ_H, ρ_L,are high and low space charges at the outside M/O (R_O) and inside O/E (R_I) interfaces respectively.

Figure S13. Schematics of the 2D axisymmetric geometry and space charge density definitions. Geometry is not drawn to scale to more clearly indicate the relevant domains. The red line represents the nanochannel centerline as axis of symmetry. Two large reservoirs (domain D4 & D5) are included on each side. Domain D2 is introduced between domain D1 and domain D3 to define a gradual and continuous transition from the high SCD gradient to the bulk, instead of abrupt changes. The length of D2 is about three times of the BOL thickness ($3R_O$)

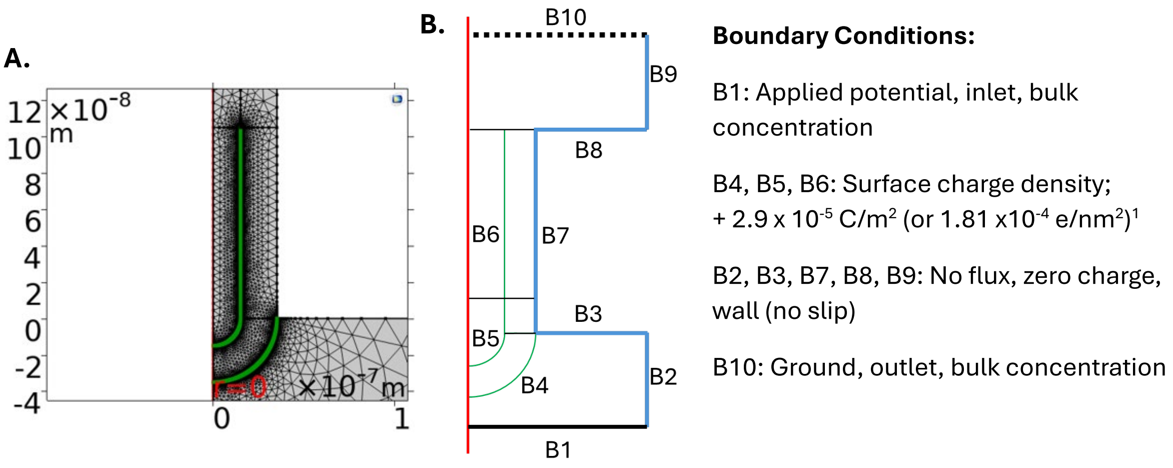

Figure S14. Adaptive mesh element definition (A) and boundary conditions (B).
Customized adaptive mesh sizes are defined as 6.25 nm to 1.85 mm with growth rate 1.25 at the M/O and O/E.

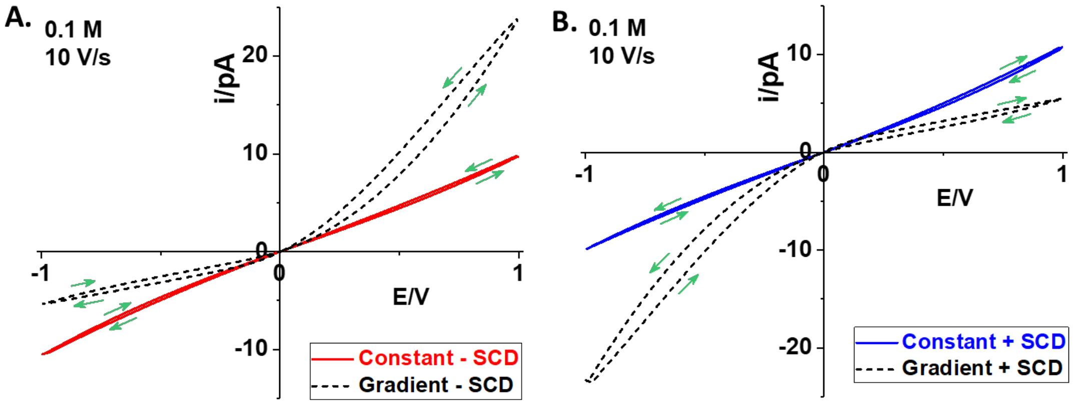

Figure S15. Simulated *I-V* curves with constant space charge density (SCD) across BOL (A) negative and (B) positive.
I-V plot of AAOs with negative (red) and positive (blue) constant space charge density (± 1.6×10^6 C/m^3) across BOL. Those in Fig. 6 with SCD gradient are also included as dashed lines for direct comparison. Green arrows indicate potential scanning direction. ICR disappears under a constant space charge regardless of positive or negative space charge polarity.

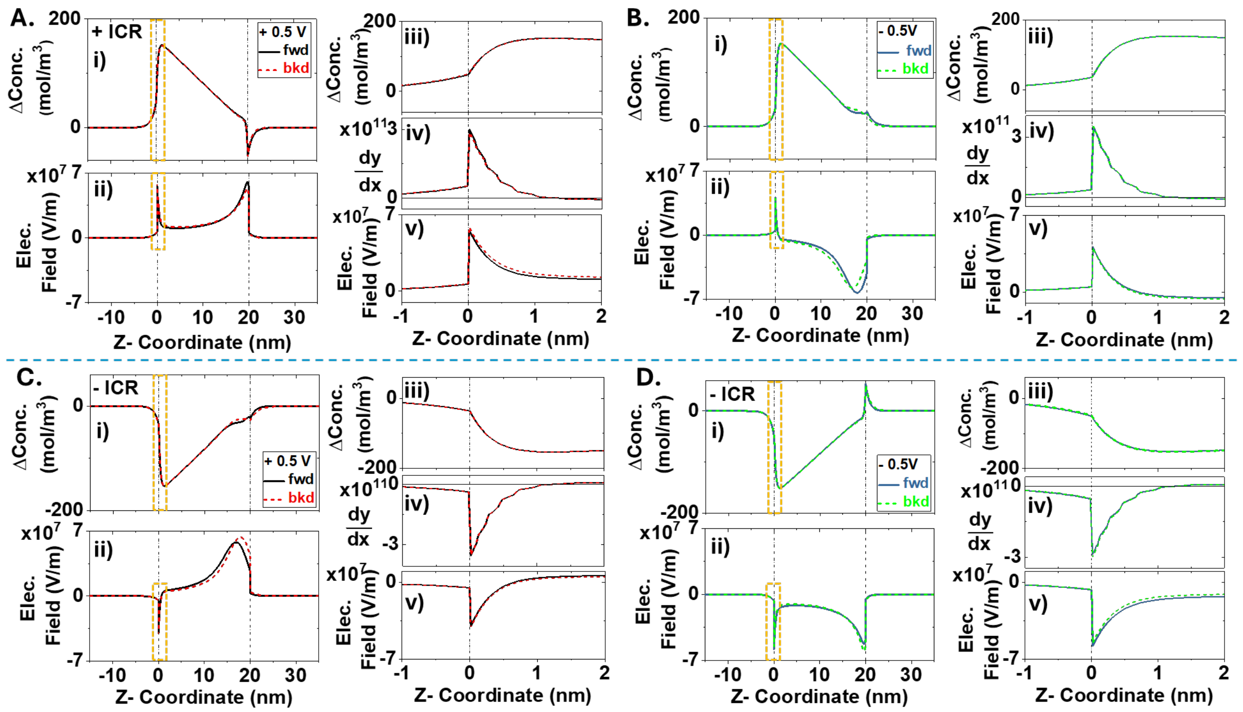

Figure S16: Electric field, concentration difference (Δ conc.) and its gradient ($\frac{dy}{dx}$ i.e., $\frac{dC}{dz}$).

Upper panel for + ICR in A. + 0.5V and B. - 0.5V; bottom panel for -ICR in C. + 0.5V and D. – 0.5V**.** i) concentration difference (Δ conc.= $[K^+]$ – $[Cl^-]$), ii) electric field across BOL. iii), iv) and v) are zoomed in (orange color box) near M/O (0 nm) of concentration difference, derivative of concentration difference and electric field respectively.

References:

1. Kim, Y. D.; Choi, S.; Kim, A.; Lee, W., Ionic Current Rectification of Porous Anodic Aluminum Oxide (AAO) with a Barrier Oxide Layer. *ACS Nano* **2020,** *14* (10), 13727-13738.
2. Parkoła, G. D. S. K. G., Temperature influence on well-ordered nanopore structures grown by anodization of aluminium in sulphuric acid. *Electrochimica Acta* **2007,** *52* (5), 1880–1888.
3. Prasad Ojha, G.; Muthurasu, A.; Prasad Tiwari, A.; Pant, B.; Chhetri, K.; Mukhiya, T.; Dahal, B.; Lee, M.; Park, M.; Kim, H.-Y., Vapor solid phase grown hierarchical CuxO NWs integrated MOFs-derived CoS2 electrode for high-performance asymmetric supercapacitors and the oxygen evolution reaction. *Chemical Engineering Journal* **2020,** *399*, 125532.
4. Krylova, V.; Andrulevičius, M., Optical, XPS and XRD Studies of Semiconducting Copper Sulfide Layers on a Polyamide Film. *International Journal of Photoenergy* **2009,** *2009* (1), 304308.
5. Fantauzzi, M.; Elsener, B.; Atzei, D.; Rigoldi, A.; Rossi, A., Exploiting XPS for the identification of sulfides and polysulfides. *RSC Advances* **2015,** *5* (93), 75953-75963.
